# Trifluridine–tipiracil plus bevacizumab versus trifluridine–tipiracil monotherapy for chemorefractory metastatic colorectal cancer: a systematic review and meta-analysis

**DOI:** 10.1186/s12885-024-12447-8

**Published:** 2024-06-03

**Authors:** Francisco Cezar Aquino de Moraes, Felipe Dircêu Dantas Leite Pessôa, Caio Henrique Duarte de Castro Ribeiro, Marianne Rodrigues Fernandes, Rommel Mario Rodríguez Burbano, Ney Pereira Carneiro dos Santos

**Affiliations:** 1 Oncology Research Center, Federal University of Pará, University Hospital João de Barros de Barreto. Rua dos Mundurucus, nº4487, Belem, 66073-005 PA Brazil; 2 School of Medicine, University of São Paulo – USP, São Paulo, 01246-903 Brazil; 3 School of Medicine, Federal University of Pará, Belem, 66075-110 Brazil; 4 Ophir Loyola Hospital, Belém, 66063-240 PA Brazil

**Keywords:** Colorectal cancer, Trifluridine-tipiracil, Bevacizumab, anti-VEGF antibody

## Abstract

Colorectal cancer is the leading cause of cancer death worldwide. The first and second lines of treatment for metastatic colorectal cancer (mCRC) include chemotherapy based on 5-fluorouracil. However, treatment following progression on the first and second line is still unclear. We searched PubMed, Scopus, Cochrane, and Web of Science databases for studies investigating the use of trifluridine-tipiracil with bevacizumab versus trifluridine-tipiracil alone for mCRC. We used RStudio version 4.2.3; and we considered *p* < 0.05 significant. Seven studies and 1,182 patients were included − 602 (51%) received trifluridine-tipiracil plus bevacizumab. Compared with control, the progression-free survival (PFS) (HR 0.52; 95% CI 0.42–0.63; *p* < 0.001) and overall survival (OS) (HR 0.61; 95% CI 0.52–0.70; *p* < 0.001) were significantly higher with bevacizumab. The objective response rate (ORR) (RR 3.14; 95% CI 1.51–6.51; *p* = 0.002) and disease control rate (DCR) (RR 1.66; 95% CI 1.28–2.16; *p* = 0.0001) favored the intervention. Regarding adverse events, the intervention had a higher rate of neutropenia (RR 1.38; 95% CI 1.19–1.59; *p* = 0.00001), whereas the monotherapy group had a higher risk of anemia (RR 0.60; 95% CI 0.44–0.82; *p* = 0.001). Our results support that the addition of bevacizumab is associated with a significant benefit in PFS, OS, ORR and DCR.

**Graphical Abstract:**

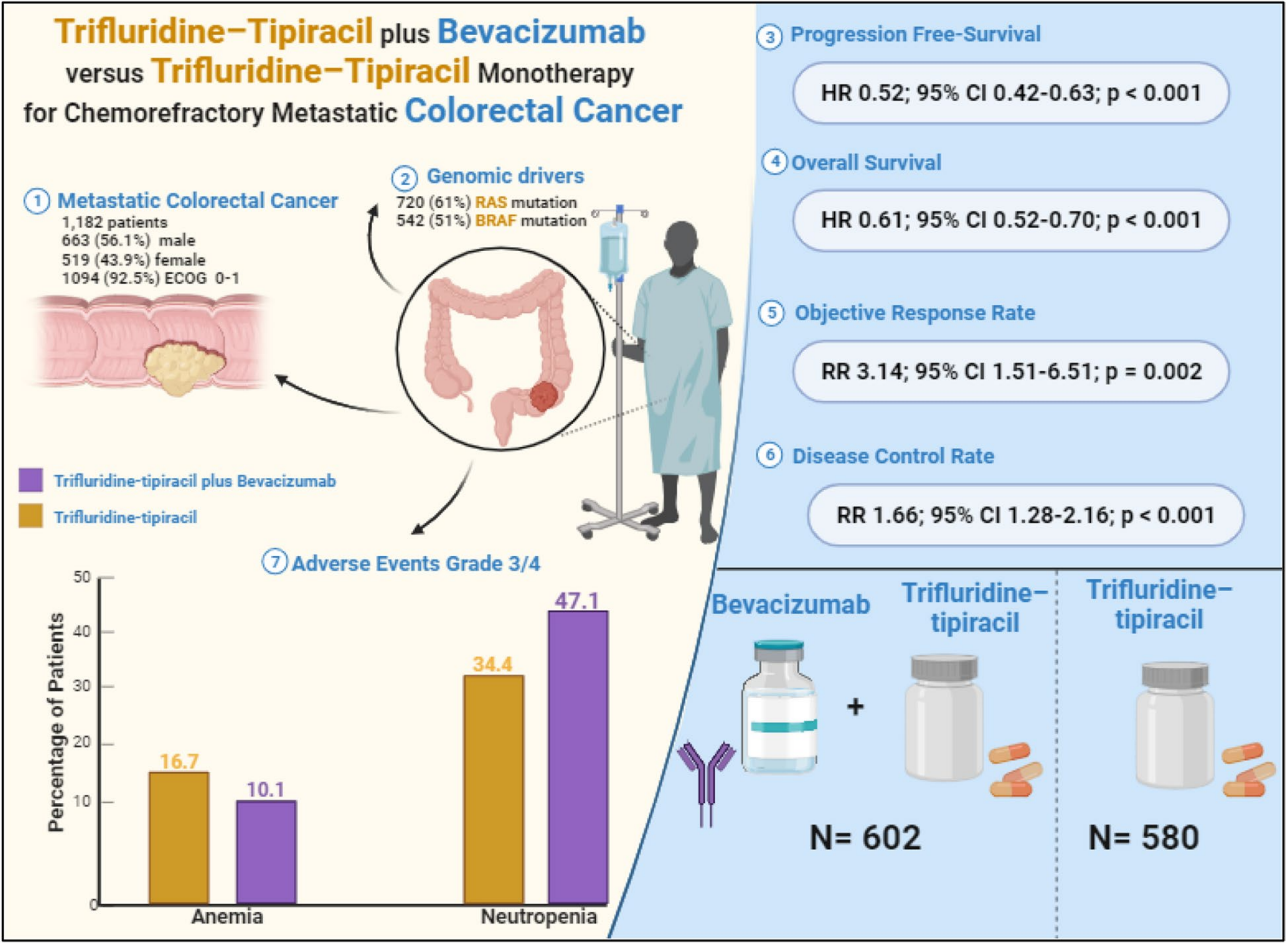

**Supplementary Information:**

The online version contains supplementary material available at 10.1186/s12885-024-12447-8.

## Introduction

Colorectal cancer (CRC) is one of the leading causes of cancer death, accounting for one in 10 cases, with an estimated 1,9 million new cases per year worldwide [1, 2]. Generally, first- and second-line treatment consists of fluorouracil-based chemotherapy with oxaliplatin and irinotecan, therapy targeting the vascular endothelial growth factor (VEGF) (mainly with Bevacizumab) or the epidermal growth factor receptor (EGFR) (the latter mainly in RAS wild-type tumors) [3–6]. When disease progression occurs after these therapies, patients are considered chemorefractory; however, as many of these patients perform well for treatment, they may be eligible for additional therapies, as progression-free survival is less than 2 months without additional therapy [7–10].

Trifluridine/tipiracil is an orally administered combination of trifluridine, a nucleic acid analog, and tipiracil, a thymidine phosphorylase inhibitor [11, 12]. Trifluridine is an active cytotoxic component that, inside neoplastic cells, is phosphorylated by thymidine kinase to form trifluridine triphosphate, which acts by incorporating itself into the cell DNA in place of thymine [13]. Thymidine phosphorylase is the enzyme responsible for the metabolism of trifluridine in the liver and gastrointestinal tract, transforming it into inactive forms; however, the addition of tipiracil to the combination is responsible for the total inhibition of this degradation, thus increasing the bioavailability of trifluridine [14, 15].

Continuous inhibition of angiogenesis, particularly with anti-VEGF antibodies, is an effective strategy for treating metastatic CRC [16, 17]. Bevacizumab, an anti-VEGF antibody, improved progression-free survival and overall survival in patients with metastatic CRC when added to first- or second-line chemotherapy [18]. More interestingly, the phase I/II C-TASK FORCE [19] study showed promising anti-tumor activity of TAS-102 (trifluridine/tipiracil) with bevacizumab in 25 colorectal cancer patients refractory to standard therapy. In this study, the median progression-free survival (PFS) was 5.6 months (95% CI; 3.4–7.6) and the median overall survival was 5.6 months (95% CI; 7.6–13.9). In contrast, these data are promising compared with those of large randomized trials that evaluated TAS-102 in monotherapy [20–23].

Thus, this meta-analysis clarified the real benefit of adding bevacizumab to trifluridine/tipiracil when compared directly with trifluridine/tipiracil in patients with chemorefractory metastatic CRC.

## Methods

### Protocol and registration

This systematic review adhered rigorously to the guidelines established by the Cochrane Collaboration and the Preferred Reporting Items for Systematic Reviews and Meta-Analyses (PRISMA) (Supplementary Tables 1 and 2) [24, 25]. To ensure transparency and reduce the risk of bias, the protocol was prospectively registered in the International Prospective Register of Systematic Reviews (PROSPERO) under the registration number CRD42024498571.

The studies were selected on the basis of the PICOT question, including studies in patients with chemorefractory metastatic colorectal cancer (P-population) taking bevacizumab plus trifluridine/tipiracil (I-intervention) or trifluridine/tipiracil monotherapy (C-control) to evaluate efficacy and safety (O-outcome). Thus, we sought to answer the following question: the addition of bevacizumab to trifluridine/tipiracil is effective and safe?

### Eligibility criteria

Studies that met the following eligibility criteria were included: (1) clinical case-control and cohort studies; (2) trifluridine/tipiracil (35 mg/m² of body surface area) orally twice a day on days 1–5 and 8–12 in a 28-day cycle with or without bevacizumab (5 mg/kg of body weight) administered by intravenous infusion every 2 weeks; (3) patients ≥ 18 years of age with metastatic colorectal cancer; (4) refractory to fluoropyrimidine, irinotecan, and oxaliplatin; and (5) patients who have progressed to at least 1 line of treatment. We excluded studies with overlapping populations, case reports, reviews, editorials, conference abstracts, and studies with no outcomes of interest. Inclusion and exclusion criteria for the studies included in the systematic review and meta-analysis are detailed in Table S3.

### Search strategy

PubMed, Cochrane Library, Scopus, and Web of Science were systematically searched on December 17, 2023. The detailed search strategy, utilizing MeSH terms, is provided in Table S4 of the Supplementary Material. To maximize capture of relevant studies, we went beyond the initial database search. Two reviewers (F.C.A.M. and F.D.D.L.P.) independently assessed the references of included articles and past systematic reviews. Additionally, we set up alerts in each database to automatically notify us of any newly published studies relevant to our inquiry. All identified articles, both from databases and reference lists, were imported into EndNote® X7 (Thomson Reuters, Philadelphia, USA) for reference management. We employed a combined approach of automated and manual methods to meticulously remove duplicate entries. Subsequently, both reviewers independently screened the titles and abstracts of retrieved articles. Should any discrepancies arise, consensus was achieved through discussions involving the two reviewers and the senior author (N.P.C.S.).

### Data extraction

The following baseline characteristics were extracted: (1) ClinicalTrials.gov Identifier; (1) study design; (3) regimen details in the intervention and control arm (Supplementary Table S5); (4) number of patients allocated for each arm; and (5) main patient characteristics. The ensuing outcomes of interest were extracted: (1) PFS, defined as the time from patient randomization to disease progression or death from any cause; (2) OS, defined as the time from the start of treatment that patients are still alive; (3) Disease control rate (DCR), defined as the sum of complete response (CR), partial response (PR) and stable disease (SD); (4) Objective response rate (ORR), defined as the sum of CR and PR [26]; and (5) adverse events, defined as an unwanted effect of a treatment, which were evaluated by the Common Terminology Criteria for Adverse Events, version 5.0 [27]. Two authors (C.H.D.C.R. and F.D.D.L.P.) collected pre-specified baseline characteristics and outcome data. Where available, the full protocol of each study was consulted to verify study objectives, population, and other relevant information regarding study design and conduction. For publications reporting results from the same study, the most recent or complete publication reporting the information of interest was considered.

### Endpoints and subgroup analysis

Outcomes of interest included: (1) PFS; (2) OS; (3) ORR; (4) DCR and patients with grade ≥ 3 of (5) neutropenia; (6) anemia; (7) thrombocytopenia; (8) nausea; (9) diarrhea; (10) vomiting; (11) fatigue and (12) febrile neutropenia.

### Risk of bias assessment

To ensure objectivity and minimize individual bias, three independent reviewers (F.D.D.L.P., C.H.D.C.R., and F.C.A.M.) evaluated the risk of bias within each included randomized controlled trial. Any discrepancies were resolved through consensus discussions to achieve a unified judgment. The Cochrane Collaboration tool for assessing risk of bias in randomized trials (RoB 2) was utilized for quality assessment of individual randomized studies [28]. Each trial was assigned a score of high, low, or unclear risk of bias across five domains: randomization process, deviations from intended interventions, missing outcomes, measurement of outcomes, and selection of reported results. Non-randomized interventional studies were assessed through the Risk Of Bias In Non-randomized Studies of Interventions (ROBINS-I) tool [29], which contains seven domains and categorizes studies as having low, moderate, serious, critical, or unclear risk of bias. Funnel-plot analyses were employed to examine publication bias [30].

### Statistical analysis

For time-to-event outcomes like progression-free survival (PFS) and overall survival (OS), we utilized the hazard ratio (HR) as the primary measure of effect. Higher HRs (> 1) favored the control group, indicating a greater risk of the event occurring in that group compared to the intervention group. Conversely, HRs less than 1 indicated a benefit associated with the intervention. For outcomes with binary endpoints, we employed risk ratios (RRs) alongside their corresponding 95% confidence intervals (CIs). These provided the relative risk of experiencing the event in one group compared to the other [31]. The Sidik-Jonkman estimator was used to calculate the tau2 variance between studies [32]. We used DerSimonian and Laird random-effect models for all endpoints [27]. Publication bias was explored using Egger’s linear regression test [33]. The packages used were “meta” and “metagen”. Statistical analyses were performed using R statistical software, version 4.2.3 (R Foundation for Statistical Computing).

## Results

### Search results and characteristics of included studies

**Fig. 1:**
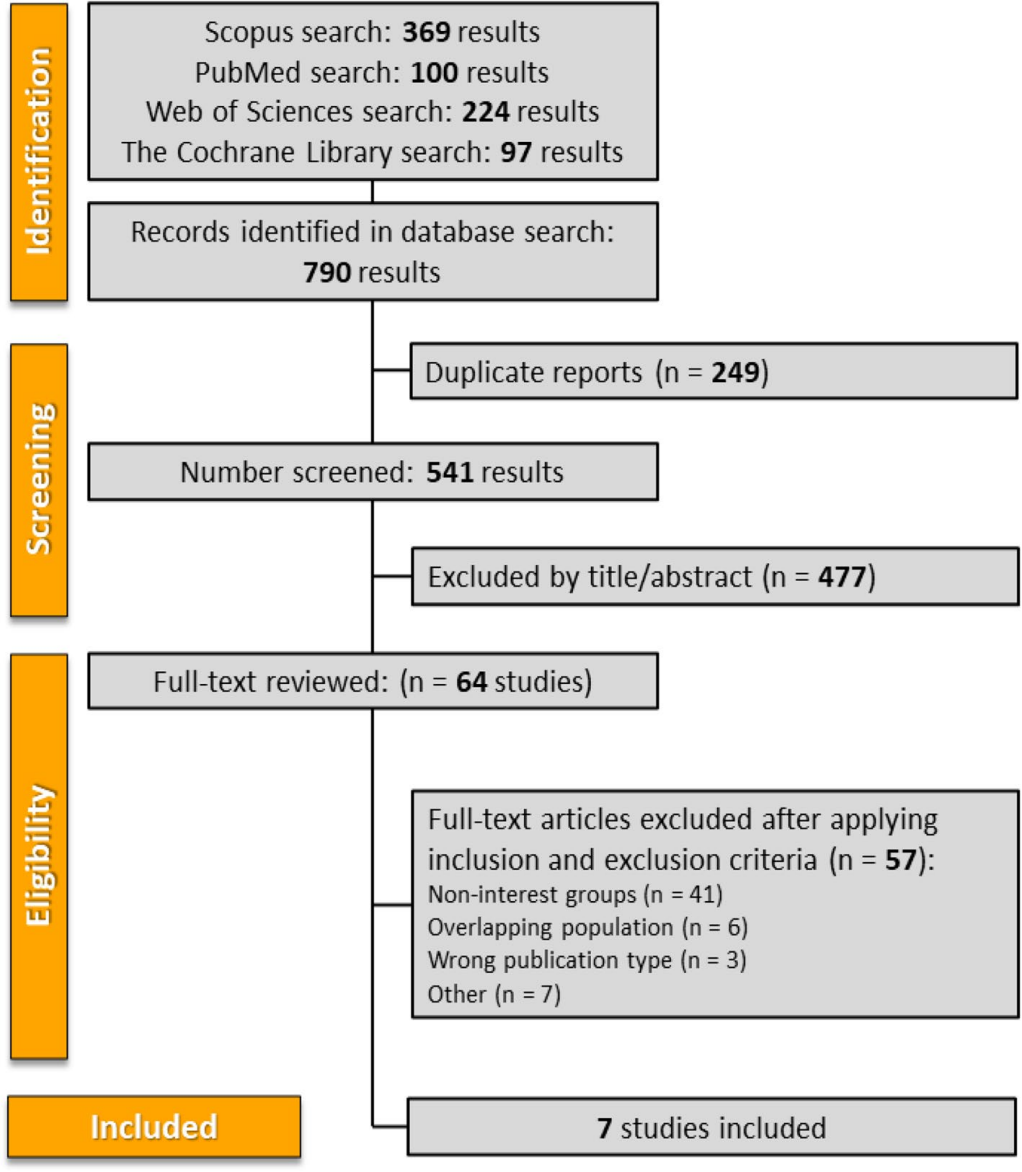
PRISMA flow diagram of study screening and selection

The selection process is shown in detail in a PRISMA flow diagram (Fig. 1). Our systematic search identified a total of 790 references. After removing 249 duplicates and screening titles and abstracts for eligibility, we excluded 477 references and assessed 64 full-text manuscripts for inclusion and exclusion criteria. Of these, seven studies [34–40] met the criteria and were included in the analysis: two clinical trials and five retrospective cohort studies. These seven studies comprised a total of 1,182 patients.

A total of 602 patients with colorectal cancer were randomized to trifluridine-tipiracil plus bevacizumab and 580 patients to trifluridine-tipiracil monotherapy. The baseline characteristics of the included studies are summarized in Table 1. The median age ranged from 20 to 90 years. 663 (56.1%) patients were male and 519 (43.9%) were female. 1094 (92.5%) had an ECOG performance status of 0 or 1 and 31 (2,62%) had an ECOG ≥ 2. The primary tumor site of 683 (57.7%) patients was the left side and for 433 (36.6%) patients was the right side. 715 (60.5%) had one or two metastatic sites. The liver was affected in 305 (25.8%) patients, lung in 283 (23.9%), peritoneum in 100 (8.4%), lymph nodes in 71 (6.0%), and other sites in 38 (3.2%). RAS mutant-type was present in 720 (61%) patients and wild-type in 327 (27.6%). BRAF mutant-type was present in 39 (3.3%) patients and wild-type in 542 (45.8%). At least 1,063 (89.9%) patients received prior therapy with fluoropyrimidines, 1,054 (89.2%) received oxaliplatin; 1055 (89.3%) received irinotecan; 873 (73,9%) received at least one anti-VEGF agent, and 344 (29,1%) received at least one anti-EGFR agent in their previous treatment. The characteristics of the patients are summarized in Table 1 and Supplementary Table S6.

**Table 1:** Baseline characteristics of included studies

Study	PFEIFFER et al., 2020		CHIDA et al., 2021		PRAGER et al., 2023		KOTANI et al., 2019		FUJII et al., 2019		SHIBUTANI et al., 2020		NIE et al., 2023	
***Design***	RCT - Phase II		RCS		RCT		RCS		RCS		RCS		RCS	
***Group***	I *n* = 46	C *n* = 47	I *n* = 139	C *n* = 153	I *n* = 246	C *n* = 246	I *n* = 60	C *n* = 66	I *n* = 21	C *n* = 36	I *n* = 36	C *n* = 26	I *n* = 54	C *n* = 6
***Follow-up***	10		25.3		14.2	13.6	7.1	7.2	14.8		NA		NA	
***Age***	64 (57–69)	67 (58–72)	61 (50–70)	65 (58–71)	62 (20–84)	64 (24–90)	60 (23–79)	65 (30–80)	67 (50–74)	67.5 (59.8–71.2)	68 (44–88)	69 (24–89)	55 (32–86)	59 (37–67)
***Male Sex (%)***	24 (52.1)	30 (63.8)	87 (62.6)	92 (60.1)	122 (49.6)		35 (58.3)	42 (63.6)	13 (61.9)	16 (44.4)	15 (41.6)	21 (80.7)	29 (53.7)	3 (50.0)
***PS-ECOG (%)***	**0, 1**–46 (100) **≥2 −** 0 (0)	**0, 1**–47 (100) **≥2** − 0 (0)	**0, 1–137** (98.6) **≥2**–2 (1.4)	**0, 1–148** (96.7) **≥2**–5 (3.3)	**0, 1–246** (100) **≥2 − 0 (0)**	**0, 1–245** (99.6) **≥2** − 1 (0.4)	**0, 1**–59 (98.3) **≥2** − 1 (1.7)	**0, 1**–63 (95.4) **≥2**–3 (4.5)	NA		**0, 1**–35 (97.2) **≥2** − 1 (2.7)	**0, 1**–21 (80.8) **≥2**–5 (19.2)	**0, 1**–43 (79.6) **≥2**–11 (20.4)	**0, 1**–4 (66.7) **≥2**–2 (33.3)
***Primary tumor location (%)***	**RS / Colon** − 11 (23.9) **LS / Rectum** − 35 (76)	**RS / Colon** − 11 (23.4) **LS / Rectum** − 36 (76.5)	**RS / Colon** − 30 (21.6) **LS / Rectum** − 109 (78.4)	**RS / Colon** − 32 (20.9) **LS / Rectum** − 121 (79.1)	**RS / Colon** − 122 (49.6) **LS / Rectum** − 124 (50.4)	**RS / Colon** − 134 (54.5) **LS / Rectum** − 112 (45.5)	**RS / Colon** − 11 (18.3) **LS / Rectum** − 49 (81.70)		**RS / Colon** − 14 (66.6) **LS / Rectum** − 7 (33.3)	**RS / Colon** − 14 (38.9) **LS / Rectum** − 22 (61.1)	**RS / Colon** − 13 (36.1) **LS / Rectum** − 23 (63.9)	**RS / Colon** − 7 (26.9) **LS / Rectum** − 19 (73.1)	**RS / Colon** − 31 (57.4) **LS / Rectum** − 23 (42.6)	**RS / Colon** − 3 (50.0) **LS / Rectum** − 3 (50.0)
***RAS mutant-type (%)***	27 (58.7)	29 (61.7)	76 (54.7)	91 (59.5)	171 (69.5)	170 (69.1)	32 (53.3)	36 (54.5)	11 (52.4)	20 (55.6)	16 (44.4)	10 (38.4)	29 (53.7)	2 (33.3)
***BRAF mutant-type (%)***	2 (4.3)	0 (0)	5 (3.6)	7 (4.6)	8 (3.3)	11 (4.5)	1 (1.7)	4 (6.1)	NA		NA		1 (1.9)	0 (0)
***Median overall survival, months (95% CI)***	9.4 months (7.6–10.7)	6.7 months (4.9–7.6)	11.5 months (9.9–13.9)	8.1 months (6.8–9.2)	10.8 months (9∙4–12∙1)	7.5 months (6.3–8.6)	8.6 months (6.9–10.3)	8.0 months (6.6–9.4)	14.4 months (7.9–NA)	4.5 months (3.2–6.5)	NA		12.0 months (9.6–14.4)	6.5 months (0.1–12.9)
***Median progression-free survival, months (95% CI)***	4.6 months (3.5–6.5)	2.6 months (1.6–3.5)	4.4 months (3.7–5.4)	2.5 months (2.1–3.1)	5.6 months (4.5–5.9)	2.4 months (2.1–3.2)	3.7 months (2.3–5.1)	2.2 months (1.8–2.6)	NA		NA		6.3 months (5.2–7.4)	3.0 months (2.2–3.8)

*RCT* Randomized Controlled Trial, *RCS* Retrospective Cohort Study, *I* Intervention group, *C* Control group, *PS-ECOG* Performance Status of Eastern Cooperative Oncology Group, *RS* Right side, *LS* Left side, *No* Number, *CI* Confidence Interval

### Results based on outcome

#### Progression-free survival

**Fig. 2:**
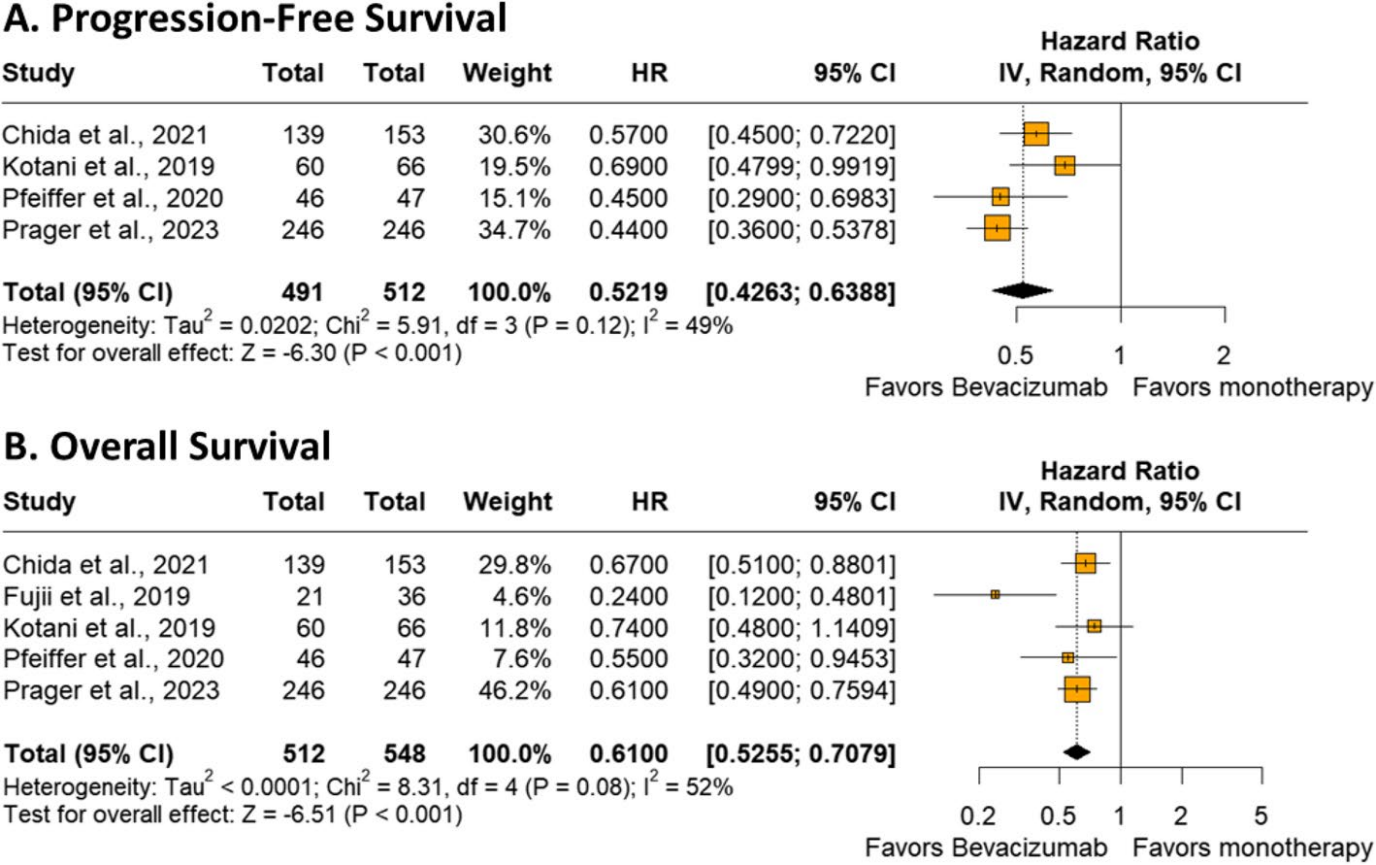
**A **Progression-free survival of patients with colorectal cancer treated with trifluridine-tipiracil plus bevacizumab versus trifluridine-tipiracil monotherapy. **B **Overall survival of patients with chemorefractory metastatic colorectal cancer treated with trifluridine-tipiracil plus bevacizumab versus trifluridine-tipiracil monotherapy

Among the 1,003 patients with metastatic colorectal cancer included in four studies, the estimated PFS significantly favored the trifluridine-tipiracil plus bevacizumab group (HR 0.52; 95% CI 0.42–0.63; *p* < 0.001; I²=49%; Fig. 2A).

#### Overall survival

Among 1,060 patients with chemorefractory metastatic colorectal cancer included from five studies, there was a significant difference from baseline in favor of the intervention with trifluridine/tipiracil plus bevacizumab group (HR 0.61; 95% CI 0.52–0.70; *p* < 0.001; I²=52%; Fig. 2B).

### Objective response rate

**Fig. 3:**
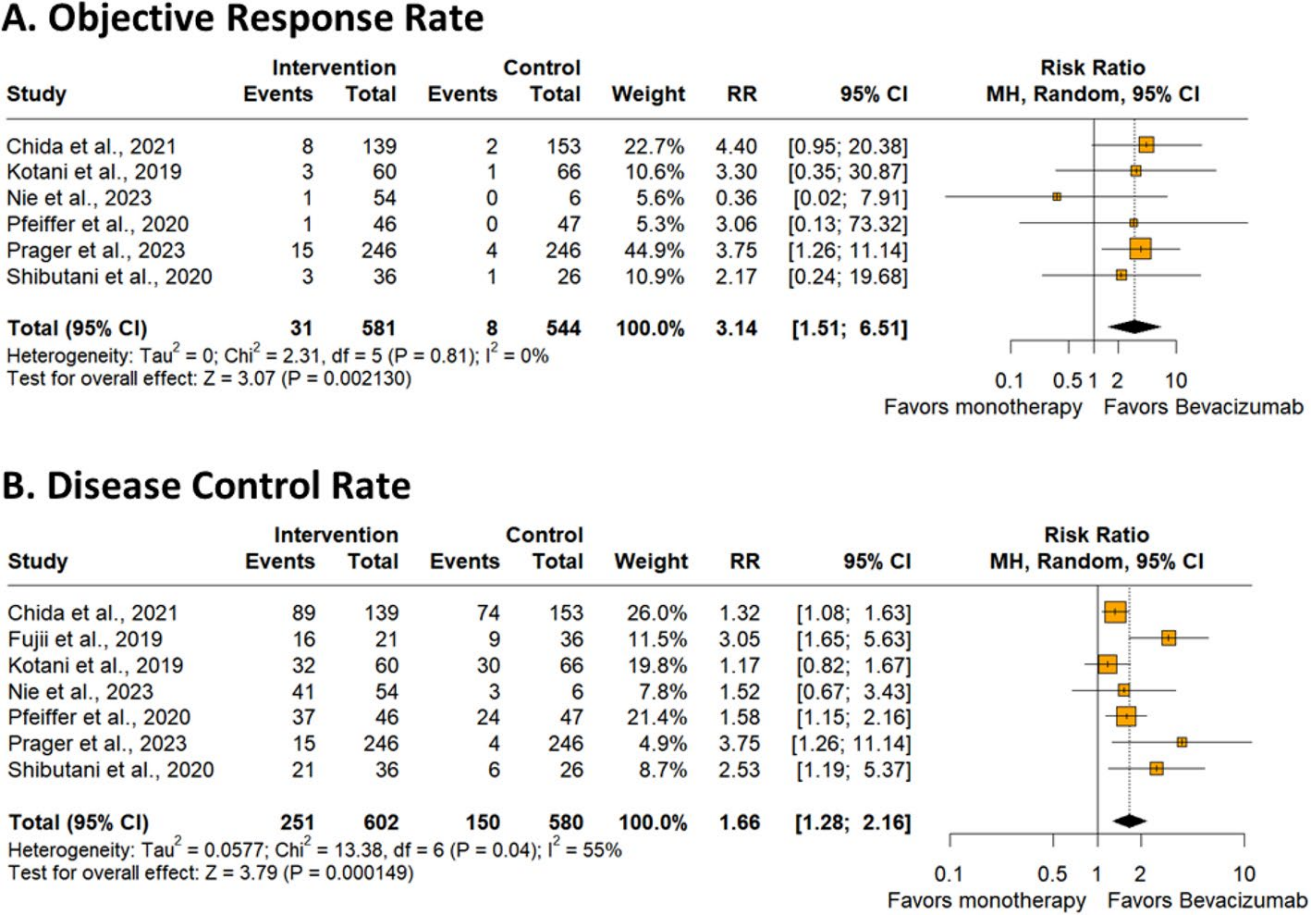
**A **Objective response rate (ORR) of patients with chemorefractory metastatic colorectal cancer treated with trifluridine-tipiracil plus bevacizumab versus trifluridine-tipiracil monotherapy. **B **Disease control rate (DCR) of patients with colorectal cancer treated with trifluridine-tipiracil plus bevacizumab versus trifluridine-tipiracil monotherapy

Six studies were incorporated with a total of 1,125 patients. The intervention group with trifluridine-tipiracil plus bevacizumab exhibited a statistically significant advantage (RR 3.14; 95% CI 1.51–6.51; *p* = 0.002; I²=0%; Fig. 3A).

### Disease control rate

Seven studies were incorporated with a total of 1,182 patients. A statistically significant superiority was observed for the bevacizumab intervention group (RR 1.66; 95% CI 1.28–2.16; *p* < 0.001; I²=55%; Fig. 3B).

#### Safety

Bevacizumab plus trifluridine-tipiracil significantly increased grade ≥ 3 of neutropenia (RR 1.38; 95% CI 1.19–1.59; *p* = 0.00001; I²=0%; Fig. S1A). In addition, trifluridine-tipiracil in monotherapy significantly increased grade ≥ 3 of anemia (RR 0.60; 95% CI 0.44–0.82; *p* = 0.001; I²=0%; Fig. S1H). There was no significant difference between the groups for grade ≥ 3 of diarrhea (RR 0.56; 95% CI 0.15–2.04; *p* = 0.37; I²=21%; Fig. S1D), fatigue (RR 0.50; 95% CI 0.20–1.23; *p* = 0.13; I²=10%; Fig. S1F), febrile neutropenia (RR 0.53; 95% CI 0.21–1.37; *p* = 0.19; I²=9%; Fig. S1G), nausea (RR 0.62; 95% CI 0.24–1.56; *p* = 0.30; I²=0%; Fig. S1C), thrombocytopenia (RR 1.48; 95% CI 0.72–3.04; *p* = 0.29; I²=0%; Fig. S1B), and vomiting (RR 0.75; 95% CI 0.25–2.21; *p* = 0.59; I²=0%; Fig. S1E). The hematological and non-hematological grade 3/4 adverse events are summarized in Table 2.

**Table 2:** Statistical analysis of the adverse events

Adverse Events Grade 3/4	No. of patients		RR	95% CI	*p*-value	Heterogeneity				
	Events/Total Intervention	Events/Total Control				Chi²	df	*p*-value	I² (%)	Tau²
Hematological toxicity										
Anemia	59/581	91/544	0.6	0.44–0.82	0.001529	2.35	5	0.8	0	0
Neutropenia	284/602	200/580	1.38	1.19–1.59	0.00001	3.51	6	0.74	0	0
Thrombocytopenia	19/581	12/544	1.48	0.72–3.04	0.290662	2.13	4	0.71	0	0
**Non-haematological toxicity**										
Diarrhea	8/521	9/478	0.56	0.15–2.04	0.37925	5.08	4	0.28	21	0.4705
Fatigue	9/467	17/472	0.5	0.2–1.23	0.130416	3.32	3	0.34	10	0.0871
Febrile neutropenia	8/281	16/292	0.53	0.21–1.37	0.190597	3.3	3	0.35	9	0.09
Nausea	9/521	10/478	0.62	0.24–1.56	0.305954	3.04	4	0.55	0	0
Vomiting	8/485	6/452	0.75	0.25–2.21	0.599817	2.37	3	0.5	0	0

*RR* Risk ratio, *CI* Confidence interval, *No* Number

### Sensitivity analyses

A leave-one-out sensitivity analysis was conducted focusing on progression-free survival (PFS), overall survival (OS), objective response rate (ORR), and disease control rate (DCR). The majority of outcomes exhibited low heterogeneity: ORR, anaemia, neutropenia, thrombocytopenia, nausea, and vomiting all demonstrated an I² of 0%; diarrhea exhibited an I² of 21%, fatigue an I² of 10%, and febrile neutropenia an I² of 9%. However, significant heterogeneity was observed in OS (I²=52%), PFS (I²=49%), and DCR (I²=55%). For OS, a notable reduction in heterogeneity was achieved by omitting the study by Fujii (2019) (HR 0.64; 95% CI 0.55–0.74; I²=0%; Fig. S2B). For PFS, a significant reduction in heterogeneity was observed upon the exclusion of Prager (2023) (HR 0.57; 95% CI 0.48–0.69; I²=8%; Fig. S2A). Nonetheless, no significant reduction in heterogeneity was noted when any of the analyzed studies were omitted for DCR (Fig. S2D).

### Quality assessment

**Fig. 4:**
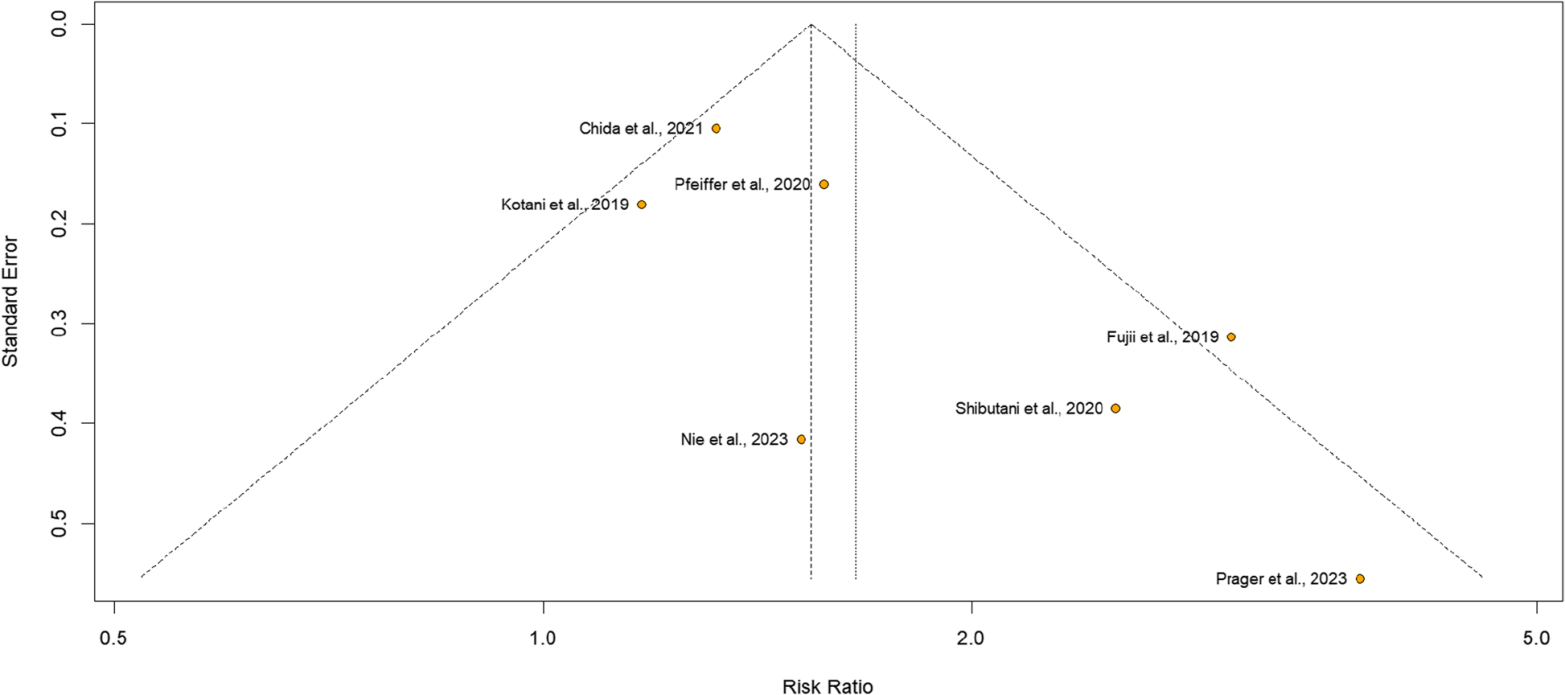
Funnel plot analysis of the disease control rate of patients with metastatic colorectal cancer

The individual assessment of each studies included in the meta-analysis is depicted in Figure S4. The analysis of the RCTs showed a low risk of bias. In the analysis of ROBINS-I for the non-randomized studies, only Kotani et al. (2019) and Fujii et al. (2020) showed moderate reliability, specifically in domains D1 and D6, respectively (Fig. S4C). The DCR funnel plot shows a low risk of bias for most of the included studies (Fig. 4).

## Discussion

In this systematic review and meta-analysis involving 7 studies and 1,182 patients, we compared Trifluridine-Tipiracil plus Bevacizumab versus Trifluridine-Tipiracil Monotherapy in patients with metastatic colorectal cancer. The main results of the pooled analyses were as follows: (1) PFS was better in patients receiving trifluridine-tipiracil plus bevacizumab; (2) OS showed a significant difference in favor of the trifluridine-tipiracil plus bevacizumab group; (3) Clinical responses to treatment, such as ORR and DCR, were significantly beneficial in the bevacizumab group; and (3) adverse effects such as neutropenia and anemia were observed in both treatment groups.

Our results showed that combining bevacizumab with trifluridine-tipiracil significantly improved PFS compared with trifluridine-tipiracil monotherapy (HR 0.52; 95% CI 0.42–0.63; *p* < 0.001). These results are encouraging, particularly when compared with other gastrointestinal cancers treated with anti-angiogenic agents. The study conducted by Okunaka et al. [41] showed that the addition of ramucirumab (VEGF inhibitor) to trifluridine-tipiracil versus trifluridine-tipiracil monotherapy does not show a benefit for the PFS of patients with advanced gastric cancer (HR 0.66; 95% CI 0.43–1.03; *p* = 0.059).

Overall survival was significantly higher among patients who used bevacizumab instead of monotherapy (HR 0.61; 95% CI 0.52–0.70; *p* < 0.001). Similar to this, the addition of panitumumab, an anti-epidermal growth factor receptor (EGFR) monoclonal antibody (mAb), to chemotherapy with leucovorin, 5-fluorouracil, and oxaliplatin (FOLFOX) can significantly benefit patients with RAS-wild left-sided metastatic colorectal cancer compared with FOLFOX alone. The PRIME study reported a higher OS rate for this group (HR 0. 73; 95% CI 0.57–0.93; *p* = 0.011) [42, 43].

Patients in the bevacizumab group had a higher absolute ORR, with 5.14% (31) versus 1.37% (8); (RR 3.14; 95% CI 1.51–6.51; *p* = 0.002). These results suggest that the use of anti-VEGF antibody can generate substantial clinical responses to treatment. Similarly, a meta-analysis conducted by Tian et al. [44] showed that the use of anti-EGFR antibody in chemotherapy treatment with FOLFOXIRI (fluoracil, oxaliplatin and irinotecan) results in a higher ORR rate (RR 1.33; 95% CI; 1.13–1.58; *P* = 0.0009) compared with FOLFOXIRI alone.

In addition, bevacizumab therapy had a higher absolute DCR (RR 1.66; 95% CI 1.28–2.16; *p* = 0.0001). This association signals promising prospects for metastatic colorectal cancer, where the addition of new emerging therapies does not always result in an additive benefit. Thus, contrary to our results, the meta-analysis conducted by Zeng et al. [45] showed that the use of immunotherapy, particularly immune checkpoint inhibitors, in colorectal cancer does not seem to be associated with any benefit for DCR (OR 0.97; 95% CI 0.36–2.61; *p* = 0.95).

Adverse events associated with cancer treatment affect the physical and emotional well-being and quality of life and can compromise the activities of daily living of patients with colorectal cancer [46]. Although the incidence of adverse events is higher for most combination chemotherapies, only neutropenia was associated with the addition of bevacizumab (*p* = 0.00001); more interestingly, trifluridine–tipiracil monotherapy seems to have increased anemia in severe grades (*p* = 0.001), suggesting that bevacizumab could be protective for this adverse event.

This study has some limitations. First, the analysis was mainly based on observational and non-randomized studies, which may have influenced the effect size found in our results. However, the absence of heterogeneity in the pooled analysis of most of the results suggests that our meta-analysis conveys the best available evidence. Second, the studies had different patient follow-up times, which may have affected our results. However, despite the limitations presented, this did not prevent robust conclusions on efficacy and safety outcomes showing the potential benefit of bevacizumab combined with trifluridine-tipiracil.

## Conclusion

This is the first meta-analysis to evaluate trifluridine–tipiracil plus bevacizumab versus trifluridine–tipiracil monotherapy for chemorefractory metastatic colorectal cancer. Our results support the notion that the addition of bevacizumab to trifluridine-tipiracil is associated with a significant improvement in PFS, OS, ORR, and DCR, suggesting the antitumor potential of this combination therapy.

### Supplementary Information

Supplementary Material 1.

## Data Availability

No datasets were generated or analysed during the current study.

## Abbreviations

BEV: Bevacizumab
BRAF: BRAF gene
CI: Confidence Interval
CR: Complete Response
CRC: Colorectal Cancer
DCR: Disease Control Rate
ECOG: Eastern Cooperative Oncology Group
EGFR: Epidermal Growth Factor Receptor
FOLFOX: Leucovorin, 5-Fluorouracil, Oxaliplatin chemotherapy regimen
GI: Gastrointestinal. HR: Hazard Ratio
I^2^: I squared statistic (measure of heterogeneity)
MeSH: Medical Subject Headings
MAb: Monoclonal Antibody
ORR: Objective Response Rate
OS: Overall Survival
PFS: Progression-Free Survival
PR: Partial Response
PRISMA: Preferred Reporting Items for Systematic Reviews and Meta-Analysis
PROSPERO: International Prospective Register of Systematic Reviews
RAS: RAS gene
RCT: Randomized Controlled Trial
RR: Risk Ratio
RECIST: Response Evaluation Criteria for Solid Tumors
ROBINS-I: Risk of Bias in Non-randomized Studies - 1 tool
RoB 2: Risk of Bias tool 2
USA: United States of America
VEGF: Vascular Endothelial Growth Factor
