# Supplementary material for: Trifluridine–tipiracil plus bevacizumab versus trifluridine–tipiracil monotherapy for chemorefractory metastatic colorectal cancer: a systematic review and meta-analysis
Source: BMC Cancer. 2024 Jun 3;24:674. doi: 10.1186/s12885-024-12447-8 (PMC11145814; doi:10.1186/s12885-024-12447-8)

**Supplementary Material**

**Supplementary Table 1** PRISMA 2020 Checklist.

**Supplementary Table 2** PRISMA 2020 for Abstract Checklist.

**Supplementary Table 3** Inclusion and exclusion criteria of included studies

**Supplementary Table 4** Search strategies.

**Supplementary Table 5** Treatment regimens from the randomized controlled trials included in this systematic review and meta-analysis.

**Supplementary Table 6**  Baseline characteristics of included studies.

**Supplementary Figure 1** Any grade of adverse events. **A.** Neutropenia. **B.** Thrombocytopenia. **C.** Nausea. **D.** Diarrhea**. E.** Vomiting. **F.** Fatigue**. G.** Febrile neutropenia. **H.** Anaemia. Comparison between Trifluridine-tipiracil plus Bevacizumab versus Trifluridine-tipiracil monotherapy in patients with metastatic colorectal cancer

**Supplementary Figure 2** Leave-one-out sensitivity analyses. **A.** Progression-free survival overall **B.** Overall survival. **C.** Objective response rate (ORR). **D.** Disease Control Rate (DCR).

**Supplementary Figure 3** Heterogeneity analysis. **A.1.** Heterogeneity Overall Survivor**.** **A.2.** Funnel AKT Overall Survivor. **B.1.** Heterogeneity Progression-free survival. **B.2.** Heterogeneity Progression-free survival. **C.1.** Heterogeneity Objective response rate. **C.2.** Heterogeneity Objective response rate. **D.1.** Heterogeneity Disease control rate (DCR). **D.2.** Heterogeneity Disease control rate (DCR)

**Supplementary Figure 4** Methodological quality summary using RoB2. **A.** Risk of bias domains in Randomized clinical trials (RCTs). **B.** Overall risk of bias in Randomized clinical trials (RCTs). **C.** Risk of bias domains in Retrospective cohort studies (RCS). **D.** Overall risk of bias in Retrospective cohort studies (RCS)

**Table S1. PRISMA 2020 Checklist.**

| Section and Topic | Item # | Checklist item | | Location where item is reported |
| --- | --- | --- | --- | --- |
| TITLE | | | |  |
| Title | 1 | Identify the report as a systematic review. | | Page 1 |
| ABSTRACT | | | |  |
| Abstract | 2 | See the PRISMA 2020 for Abstracts checklist. | | Supplementary Materials: Table S3. |
| INTRODUCTION | | | |  |
| Rationale | 3 | Describe the rationale for the review in the context of existing knowledge. | | Page 2 |
| Objectives | 4 | Provide an explicit statement of the objective(s) or question(s) the review addresses. | | Page 2 |
| METHODS | | | |  |
| Eligibility criteria | 5 | Specify the inclusion and exclusion criteria for the review and how studies were grouped for the syntheses. | | Pages 3 |
| Information sources | 6 | Specify all databases, registers, websites, organisations, reference lists and other sources searched or consulted to identify studies. Specify the date when each source was last searched or consulted. | | Page 3 |
| Search strategy | 7 | Present the full search strategies for all databases, registers and websites, including any filters and limits used. | | Supplementary Materials: Table S4 Search Strategies |
| Selection process | 8 | Specify the methods used to decide whether a study met the inclusion criteria of the review, including how many reviewers screened each record and each report retrieved, whether they worked independently, and if applicable, details of automation tools used in the process. | | Page 3 |
| Data collection process | 9 | Specify the methods used to collect data from reports, including how many reviewers collected data from each report, whether they worked independently, any processes for obtaining or confirming data from study investigators, and if applicable, details of automation tools used in the process. | | Page 3-4 |
| Data items | 10a | List and define all outcomes for which data were sought. Specify whether all results that were compatible with each outcome domain in each study were sought (e.g. for all measures, time points, analyses), and if not, the methods used to decide which results to collect. | | Pages 4 |
|  | 10b | List and define all other variables for which data were sought (e.g. participant and intervention characteristics, funding sources). Describe any assumptions made about any missing or unclear information. | | NO |
| Study risk of bias assessment | 11 | Specify the methods used to assess risk of bias in the included studies, including details of the tool(s) used, how many reviewers assessed each study and whether they worked independently, and if applicable, details of automation tools used in the process. | | Page 4 |
| Effect measures | 12 | Specify for each outcome the effect measure(s) (e.g. risk ratio, mean difference) used in the synthesis or presentation of results. | | Page 4 |
| Synthesis methods | 13a | Describe the processes used to decide which studies were eligible for each synthesis (e.g. tabulating the study intervention characteristics and comparing against the planned groups for each synthesis (item #5)). | | Pages 3-4 |
|  | 13b | Describe any methods required to prepare the data for presentation or synthesis, such as handling of missing summary statistics, or data conversions. | | Pages 4 |
|  | 13c | Describe any methods used to tabulate or visually display results of individual studies and syntheses. | | Page 4 |
|  | 13d | Describe any methods used to synthesize results and provide a rationale for the choice(s). If meta-analysis was performed, describe the model(s), method(s) to identify the presence and extent of statistical heterogeneity, and software package(s) used. | | Page 5 |
|  | 13e | Describe any methods used to explore possible causes of heterogeneity among study results (e.g. subgroup analysis, meta-regression). | | Pages 5 |
|  | 13f | Describe any sensitivity analyses conducted to assess robustness of the synthesized results. | | Pages 6 |
| Reporting bias assessment | 14 | Describe any methods used to assess risk of bias due to missing results in a synthesis (arising from reporting biases). | | Pages 6 |
| Certainty assessment | 15 | Describe any methods used to assess certainty (or confidence) in the body of evidence for an outcome. | | Pages 6 |
| RESULTS | | | |  |
| Study selection | 16a | Describe the results of the search and selection process, from the number of records identified in the search to the number of studies included in the review, ideally using a flow diagram. | | Page 4-6  Figure 1 |
|  | 16b | Cite studies that might appear to meet the inclusion criteria, but which were excluded, and explain why they were excluded. | NO | |
| Study characteristics | 17 | Cite each included study and present its characteristics. | | Pages 4-6  Table 1 |
| Risk of bias in studies | 18 | Present assessments of risk of bias for each included study. | | Supplementary Materials: Figure 4. |
| Results of individual studies | 19 | For all outcomes, present, for each study: (a) summary statistics for each group (where appropriate) and (b) an effect estimate and its precision (e.g. confidence/credible interval), ideally using structured tables or plots. | | Pages 6-9  Figure 2 and 3 |
| Results of syntheses | 20a | For each synthesis, briefly summarise the characteristics and risk of bias among contributing studies. | | Pages 6-9  Supplementary Materials: Figure 4 |
|  | 20b | Present results of all statistical syntheses conducted. If meta-analysis was done, present for each the summary estimate and its precision (e.g. confidence/credible interval) and measures of statistical heterogeneity. If comparing groups, describe the direction of the effect. | | Pages 6-9  Figure 2 and 3  Supplementary Materials: Figure 1, 2 and 3 |
|  | 20c | Present results of all investigations of possible causes of heterogeneity among study results. | | Page 9  Figure 4 |
|  | 20d | Present results of all sensitivity analyses conducted to assess the robustness of the synthesized results. | | Page 9-10  Supplementary Materials: Figure 2 |
| Reporting biases | 21 | Present assessments of risk of bias due to missing results (arising from reporting biases) for each synthesis assessed. | | Page NO |
| Certainty of evidence | 22 | Present assessments of certainty (or confidence) in the body of evidence for each outcome assessed. | | Page 10 |
| DISCUSSION | | | |  |
| Discussion | 23a | Provide a general interpretation of the results in the context of other evidence. | | Pages 11-12 |
|  | 23b | Discuss any limitations of the evidence included in the review. | | Page 11-12 |
|  | 23c | Discuss any limitations of the review processes used. | | Page 11-12 |
|  | 23d | Discuss implications of the results for practice, policy, and future research. | | Page 11-12 |
| OTHER INFORMATION | | | |  |
| Registration and protocol | 24a | Provide registration information for the review, including register name and registration number, or state that the review was not registered. | | Page 4 |
|  | 24b | Indicate where the review protocol can be accessed, or state that a protocol was not prepared. | | Page 4 |
|  | 24c | Describe and explain any amendments to information provided at registration or in the protocol. | | Page 4 |
| Support | 25 | Describe sources of financial or non-financial support for the review, and the role of the funders or sponsors in the review. | | Page 12 |
| Competing interests | 26 | Declare any competing interests of review authors. | | Page 12 |
| Availability of data, code and other materials | 27 | Report which of the following are publicly available and where they can be found: template data collection forms; data extracted from included studies; data used for all analyses; analytic code; any other materials used in the review. | | Page 12 |

**Table S2. PRISMA 2020 for Abstract Checklist.**

| Section and Topic | Item # | Checklist item | Reported (Yes/No) |
| --- | --- | --- | --- |
| TITLE | | |  |
| Title | 1 | Identify the report as a systematic review. | Yes |
| BACKGROUND | | |  |
| Objectives | 2 | Provide an explicit statement of the main objective(s) or question(s) the review addresses. | Yes |
| METHODS | | |  |
| Eligibility criteria | 3 | Specify the inclusion and exclusion criteria for the review. | No |
| Information sources | 4 | Specify the information sources (e.g. databases, registers) used to identify studies and the date when each was last searched. | Yes |
| Risk of bias | 5 | Specify the methods used to assess risk of bias in the included studies. | No |
| Synthesis of results | 6 | Specify the methods used to present and synthesise results. | No |
| RESULTS | | |  |
| Included studies | 7 | Give the total number of included studies and participants and summarise relevant characteristics of studies. | Yes |
| Synthesis of results | 8 | Present results for main outcomes, preferably indicating the number of included studies and participants for each. If meta-analysis was done, report the summary estimate and confidence/credible interval. If comparing groups, indicate the direction of the effect (i.e. which group is favoured). | Yes |
| DISCUSSION | | |  |
| Limitations of evidence | 9 | Provide a brief summary of the limitations of the evidence included in the review (e.g. study risk of bias, inconsistency and imprecision). | No |
| Interpretation | 10 | Provide a general interpretation of the results and important implications. | Yes |
| OTHER | | |  |
| Funding | 11 | Specify the primary source of funding for the review. | No |
| Registration | 12 | Provide the register name and registration number. | No |

**Table S3** Inclusion and exclusion criteria of included studies

| **Study** | **Inclusion Criteria** | **Exclusion Criteria** |
| --- | --- | --- |
| CHIDA et al., 2021 | 1. Clinical records of patients with mCRC who received later-line chemotherapy comprising TAS102 plus BEV, TAS102 monotherapy, or regorafenib between March 2013 and December 2019 in the National Cancer Center Hospital East (Kashiwa, Chiba, Japan). 2. Histologically conﬁrmed colorectal adenocarcinoma. 3. No prior treatment with TAS102 and regorafenib. 4. Refractory or intolerant to standard chemotherapies [FU, OX, IRI, and anti-epidermal growth factor receptor (EGFR) antibody (for KRAS/NRAS wild-type tumors)]. 5. Eastern Cooperative Oncology Group performance status (ECOG PS) of 0–2. 6. Adequate organ function. | 1. Missing follow-up information. 2. No prior irinotecan. 3. No prior oxaliplatin. 4. No prior anti-EGFR mAb in *RAS/BRAF*Wild-type. |
| FUJII et al., 2019 | 1. Data was obtained from patients’ electronic medical records in Gifu University Hospital. 2. Patients with mCRC who were refractory to fluoropyrimidine, irinotecan, oxaliplatin, anti‐VEGF therapy, and anti‐EGFR therapy (for tumors with wild‐type KRAS) who received TAS‐102 between July 2014 and December 2018. | 1. Reduction of the initial dose of TAS‐102 because of poor performance status (≥2 according to the Eastern Cooperative Oncology Group). 2. Discontinuation without image evaluation. |
| KOTANI et al., 2019 | 1. Clinical data of patients with mCRC who received trifluridine/tipiracil plus bevacizumab (who initiated from January 2016 to March 2018) or trifluridine/tipiracil monotherapy (who initiated from June 2014 to December 2015) at the National Cancer Center Hospital East. 2. Histologically confirmed colorectal adenocarcinoma. 3. No prior treatment with regorafenib. 4. Refractory or intolerant to fluoropyrimidine, oxaliplatin, and irinotecan, regardless of angiogenesis inhibitors or anti-EGFR antibody (if RAS wild-type). 5. Eastern Cooperative Oncology Group performance status (ECOG PS) 0 to 2. 6. Adequate organ function |  |
| NIE et al., 2023 | 1. Patients with metastatic colorectal cancer who had failed from 2 or more lines of prior therapy and treated with TAS-102 in the Afﬁliated Cancer Hospital of Zhengzhou University from October 2020 to February 2022 were enrolled. 2. Histopathologically and radiographically conﬁrmed metastatic colorectal cancer. 3. Progressed from at least 2 lines of standard treatment, including ﬂuorouracil, oxaliplatin, irinotecan, bevacizumab and cetuximab. 4. Based on the evaluation criteria of RECIST v1.1, presence of at least 1 measurable lesion was required. | 1. Patients who could not be assessed for clinical efﬁcacy due to receiving less than 2 cycles of TAS-102 treatment. 2. Patients with missing follow-up data were excluded. |
| PFEIFFER et al., 2020 | 1. Age ≥ 18 years. 2. Histologically verified colorectal adenocarcinoma. 3. Non-resectable mCRC. 4. Measurable or non-measurable disease. 5. Performance status (WHO) of 0-1 and a life expectancy of at least 3 months. 6. Failure of previous (or intolerance to) fluoropyrimidines, irinotecan, oxaliplatin, cetuximab or panitumumab (only for RAS wild type); prior bevacizumab, aflibercept or regorafenib allowed but not mandatory. 7. Adequate haematological function defined as neutrophils 1.5 x 10⁹/l and platelets ≥ 100 x 10⁹/l. 8. Adequate organ function (bilirubin ≤ 1.5 x UNL (upper normal limit), GFR (may be calculated) > 50 ml/min. 9. Woman of childbearing potential must have been tested negative in a serum pregnancy test within 5 days prior to randomisation. Male and female patients who have the potential to reproduce must agree to use a highly effective method of birth control. (i.e., pregnancy rate of less than 1 % per year) during the study and for 6 months after the discontinuation of study medication. 10. Has provided written informed consent prior to performance of any study procedure. 11. Written informed consent must be obtained according to the local Ethics Committee requirements. | 1. Prior history of cancer, except cervix in situ carcinoma, in situ urothelial carcinoma or previously treated and cured skin basocellular, and any other cancer in complete remission for at least 2 years. 2. Evidence of CNS metastasis, any other condition or therapy, which in the investigator’s opinion may pose a risk to the patient or interfere with the study objectives. 3. Known allergy or intolerance to any of the drugs used (trifluridine, tipiracil, or bevacizumab). |
| PRAGER et al., 2023 | 1. Male or female patient aged ≥18 years old at the time of ICF signature (or legal age depending on local country regulation). 2. Has histologically confirmed unresectable adenocarcinoma of the colon or rectum (all other histological types are excluded). 3. RAS status must have been previously determined (mutant or wild-type) based on local assessment of tumour biopsy.    1. Wild type is defined as KRAS (exon 2, 3 and 4) and NRAS (exon 2, 3 and 4) wild type.    2. Mutant is defined as at least KRAS or NRAS mutant (any exon, any mutation). 4. Has received a maximum of 2 prior chemotherapy regimens for the treatment of advanced colorectal cancer and had demonstrate progressive disease or intolerance to their last regimen.    1. Prior treatment regimens must have included a fluoropyrimidine, irinotecan, oxaliplatin, an anti-VEGF monoclonal antibody and/or an anti-EGFR monoclonal antibody for RAS wild-type patients.    2. Patients who have received adjuvant/neoadjuvant chemotherapy and had recurrence during or within 6 months of completion of the adjuvant/neoadjuvant chemotherapy can count the adjuvant/neoadjuvant therapy as one regimen of chemotherapy for advanced disease. 5. Has measurable or non-measurable disease as defined by RECIST version 1.1. 6. Is able to swallow oral tablets 7. Estimated life expectancy ≥12 weeks 8. Has an Eastern Cooperative Oncology Group (ECOG) performance status ≤1. ECOG should remain ≤1 during all the screening period (from screening visit to randomisation). 9. Has adequate organ function as defined by the following laboratory values obtained within 7 days prior to randomisation:    1. Absolute neutrophil count ≥1.5 x 10⁹/L    2. Haemoglobin ≥9 g/dL. In case of blood transfusion, the haemoglobin assessment must be performed 2 weeks or more after the transfusion.    3. Platelet count ≥100 x 10⁹/L.    4. Creatinine clearance ≥50 mL/min, assessed using the Cockcroft & Gault formula.    5. Total serum bilirubin <1.5 x upper limit of normal (ULN) (unless Gilbert disease confirmed).    6. Aspartate aminotransferase (AST; SGOT) and alanine aminotransferase (ALT; SGPT) ≤ 2.5 x ULN (unless if liver function abnormalities are due to underlying liver metastasis, AST (SGOT) and ALT (SGPT) ≤ 5 x ULN).    7. Adequate coagulation function for all patients. For patients receiving anti-coagulant therapy (except platelet antiaggregates) the adequate therapeutic levels of INR should be confirmed. 10. Female of childbearing potential must have been tested negative in a serum pregnancy test within 7 days prior to randomisation. 11. Female of childbearing potential and males with partners of childbearing potential must agree to use a highly effective method of birth control, as well as their partners lasting at least 6 months after the last dose of IMP. 12. Has provided written informed consent obtained prior to any study-specific procedure as described in Section 13.3. | 1. More than 2 prior chemotherapy regimens for the treatment of advanced colorectal cancer. 2. In the investigator’s opinion, the patient is unlikely to be compliant with the oral medication regimen or the requirements of the study for scheduled evaluations. 3. Pregnancy, lactating female, or possibility of becoming pregnant during the study. 4. Participation in another interventional study within 4 weeks prior to randomisation. Participation in study follow-up part without IMP administration, non-interventional registry or epidemiological study is allowed . 5. Patients currently receiving or having received anticancer therapies within 4 weeks prior to randomisation. 6. Already randomised in this study. 7. Has not recovered from clinically relevant non-hematologic CTCAE grade ≥ 3 toxicity of previous anticancer therapy prior to randomisation (excluding alopecia, and skin pigmentation). 8. Has symptomatic central nervous system metastases that are neurologically unstable or requiring increasing doses of steroids to control CNS disease. 9. Had major surgery within 4 weeks prior to randomisation (the surgical incision should be fully healed prior to study drug administration), or has not recovered from side effects of previous surgery, or patient that may require major surgery during the study. 10. In the investigator’s opinion, patient with chronic gastrointestinal disorders that might significantly interfere with proper absorption of the study treatments. 11. Has hereditary problems of galactose intolerance, total lactase deficiency or glucose- galactose malabsorption. 12. Has severe or uncontrolled active acute or chronic infection. 13. Has active or history of interstitial lung disease and/or pneumonitis, or pulmonary hypertension. 14. Known Hepatitis B Virus infection determined as HBsAg positive and / or known Hepatitis C Virus infection determined as detection of HCV RNA in serum or plasma by a sensitive quantitative molecular method. 15. Known carriers of HIV antibodies. 16. In the investigator’s opinion, uncontrolled diabetes mellitus even under treatment. 17. In the investigator’s opinion, uncontrolled arterial hypertension or uncontrolled or symptomatic arrhythmia. 18. Deep arterial thromboembolic events including cerebrovascular accident or myocardial infarction within the last 6 months prior to randomisation. 19. Severe/unstable angina, symptomatic congestive heart failure New York Heart Association (NYHA) class III or IV. 20. Drainage for ascites, pleural effusion or pericardial fluid within 4 weeks prior to randomisation. 21. Other malignancies including those which were radically treated and for which the remission period at the time of screening is less than five years. Exemptions for this minimally required duration of remission period may be applied for carcinoma in situ of the cervix and basal cell skin cancer that are deemed to be cured by adequate treatment. 22. Treatment with systemic immunosuppressive therapy (except steroids given in prophylactic setting or at a chronic low dose [≤20 mg/day prednisone equivalent]). 23. Prior radiotherapy if completed less than 4 weeks before randomisation, except if provided as a short course for symptoms palliation only. Tumour lesions if previously irradiated may not be chosen as target lesions for response evaluation. 24. In the investigator’s opinion, any clinically significant medical condition (e.g. organ dysfunction) or laboratory abnormality likely to jeopardize the patient’s safety or to interfere with the conduct of the study. 25. Has previously received trifluridine/tipiracil. 26. History of allergic reactions attributed to compounds of similar composition to trifluridine/tipiracil or any of its excipients. 27. Any contraindication present in the EU Product Information of trifluridine/tipiracil. 28. History of allergic reactions or hypersensitivity to bevacizumab or any of its excipients. 29. History of hypersensitivity to Chinese Hamster Ovary cell products or other recombinant human or humanised antibodies. 30. Serious non-healing wound, non-healing ulcer or non-healing bone fracture. 31. Deep venous thromboembolic event within 4 weeks prior to randomisation 32. Known coagulopathy that increases risk of bleeding, bleeding diatheses. Any other haemorrhage/bleeding event CTCAE grade ≥ 3 within 4 weeks prior to randomisation. 33. Any contraindication present in the EU Product Information of bevacizumab. |
| SHIBUTANI et al., 2020. | 1. Medical records of patients with mCRC who were treated with FTD/TPI at Osaka City University Hospital between June 2014 and February 2020. 2. Received at least two previous chemotherapeutic regimens. | 1. Patients treated with an anti-angiogenic inhibitor other than bevacizumab, such as ramucirumab and aflibercept, in front-line treatment were excluded from this study. |

**Table S4** Search Strategies

| **Database** | **Search Strategy** |
| --- | --- |
| **PubMed  100 results** | ("cancer of colon and rectum" OR "cancer of rectum and colon" OR "cancer of the colon and rectum" OR "cancer of the colon and the rectum" OR "cancer of the rectum and colon" OR "cancer of the rectum and the colon" OR "colo-rectal cancer" OR "colo-rectal carcinogenesis" OR "colo-rectal malignancies" OR "colo-rectal malignancy" OR "colorectal cancerogenesis" OR "colorectal carcinogenesis" OR "colorectal malignancies" OR "colorectal malignancy" OR "malignancies of the colon and rectum" OR "malignancy of colon and rectum" OR "malignancy of the colon and rectum" OR "recto-colonic cancer" OR "rectocolonic cancer" OR "colorectal cancer" OR “colorectal cancers” OR “Colorectal Neoplasm” OR “Colorectal Tumors” OR “Colorectal Tumor” OR “colorectal carcinoma” OR “colorectal carcinomas”) AND ("abevmy" OR "abp 215" OR "abp215" OR "ainex" OR "altuzan" OR "alymsys" OR "ankeda" OR "ask b1202" OR "askb1202" OR "avastin" OR "avegra" OR "aybintio" OR "ba 1101" OR "ba1101" OR "bambevi" OR "bat 1706" OR "bat1706" OR "bcd 021" OR "bcd021" OR "bevacizumab adcd" OR "bevacizumab awwb" OR "bevacizumab beta" OR "bevacizumab bvzr" OR "bevacizumab gamma" OR "bevacizumab maly" OR "bevacizumab-adcd" OR "bevacizumab-awwb" OR "bevacizumab-bvzr" OR "bevacizumab-maly" OR "bevagen" OR "bevatas" OR "bevax" OR "bevz 92" OR "bevz92" OR "bi 695502" OR "bi695502" OR "bow 030" OR "bow030" OR "boyounuo" OR "bp 01" OR "bp 102" OR "bp01" OR "bp102" OR "bryxta" OR "bs 503a" OR "bs503a" OR "bxt 2316" OR "bxt2316" OR "byvasda" OR "cbt 124" OR "cbt124" OR "chs 305" OR "chs 5217" OR "chs305" OR "chs5217" OR "cizumab" OR "ct p16" OR "ctp16" OR "equidacent" OR "fkb 238" OR "fkb238" OR "gb 222" OR "gb222" OR "gbs 004" OR "gbs004" OR "hanbeitai" OR "hd 204" OR "hd204" OR "hlx 04" OR "hlx04" OR "hot 1010" OR "hot1010" OR "ibi 305" OR "ibi305" OR "idb 0072" OR "idb0072" OR "intp 24" OR "intp24" OR "ipique" OR "jhl 1149" OR "jhl1149" OR "js 501" OR "js501" OR "jy 028" OR "jy028" OR "krabeva" OR "kyomarc" OR "lextemy" OR "lumiere (drug)" OR "ly 01008" OR "ly01008" OR "mabionvegf" OR "mb 02" OR "mb02" OR "mil 60" OR "mil60" OR "mvasi" OR "myl 14020" OR "myl 1402o" OR "myl14020" OR "myl1402o" OR "nsc 704865" OR "nsc704865" OR "onbevzi" OR "ons 1045" OR "ons 5010" OR "ons1045" OR "ons5010" OR "oyavas" OR "pf 06439535" OR "pf 6439535" OR "pf06439535" OR "pf6439535" OR "pmc 901" OR "pmc901" OR "pobevcy" OR "pro 169" OR "pro169" OR "pusintin" OR "ql 1101" OR "ql1101" OR "r 435" OR "r tpr 023" OR "r435" OR "rg 435" OR "rg435" OR "rhuMAb-VEGF" OR "ro 4876646" OR "ro4876646" OR "rph 001" OR "rph001" OR "rtpr023" OR "sb 8" OR "sb8" OR "sct 501" OR "sct 510" OR "sct501" OR "sct510" OR "stc 103" OR "stc103" OR "stivant" OR "tab 008" OR "tab 014" OR "tab008" OR "tab014" OR "tot 102" OR "tot102" OR "trs 003" OR "trs003" OR "tx 16" OR "tx16" OR "vegzelma" OR "versavo" OR "zirabev" OR "zrc 113" OR "zrc113" OR "zybev" OR "bevacizumab") AND ("lonsurf" OR "tas 102" OR "tas102" OR "tipiracil hydrochloride plus trifluridine" OR "tipiracil hydrochloride/trifluridine" OR "tipiracil/trifluridine" OR "trifluridine plus tipiracil" OR "trifluridine plus tipiracil hydrochloride" OR "trifluridine/tipiracil" OR "trifluridine/tipiracil hydrochloride" OR "tipiracil plus trifluridine") |
| **Scopus**  **369 results** | (TITLE-ABS-KEY("cancer of colon and rectum") OR TITLE-ABS-KEY("cancer of rectum and colon") OR TITLE-ABS-KEY("cancer of the colon and rectum") OR TITLE-ABS-KEY("cancer of the colon and the rectum") OR TITLE-ABS-KEY("cancer of the rectum and colon") OR TITLE-ABS-KEY("cancer of the rectum and the colon") OR TITLE-ABS-KEY("colo-rectal cancer") OR TITLE-ABS-KEY("colo-rectal carcinogenesis") OR TITLE-ABS-KEY("colo-rectal malignancies") OR TITLE-ABS-KEY("colo-rectal malignancy") OR TITLE-ABS-KEY("colorectal cancerogenesis") OR TITLE-ABS-KEY("colorectal carcinogenesis") OR TITLE-ABS-KEY("colorectal malignancies") OR TITLE-ABS-KEY("colorectal malignancy") OR TITLE-ABS-KEY("malignancies of the colon and rectum") OR TITLE-ABS-KEY("malignancy of colon and rectum") OR TITLE-ABS-KEY("malignancy of the colon and rectum") OR TITLE-ABS-KEY("recto-colonic cancer") OR TITLE-ABS-KEY("rectocolonic cancer") OR TITLE-ABS-KEY("colorectal cancer") OR TITLE-ABS-KEY("colorectal cancers") OR TITLE-ABS-KEY("Colorectal Neoplasm") OR TITLE-ABS-KEY("Colorectal Tumors") OR TITLE-ABS-KEY("Colorectal Tumor") OR TITLE-ABS-KEY("colorectal carcinoma") OR TITLE-ABS-KEY("colorectal carcinomas")) AND (TITLE-ABS-KEY("abevmy") OR TITLE-ABS-KEY("abp 215") OR TITLE-ABS-KEY("abp215") OR TITLE-ABS-KEY("ainex") OR TITLE-ABS-KEY("altuzan") OR TITLE-ABS-KEY("alymsys") OR TITLE-ABS-KEY("ankeda") OR TITLE-ABS-KEY("ask b1202") OR TITLE-ABS-KEY("askb1202") OR TITLE-ABS-KEY("avastin") OR TITLE-ABS-KEY("avegra") OR TITLE-ABS-KEY("aybintio") OR TITLE-ABS-KEY("ba 1101") OR TITLE-ABS-KEY("ba1101") OR TITLE-ABS-KEY("bambevi") OR TITLE-ABS-KEY("bat 1706") OR TITLE-ABS-KEY("bat1706") OR TITLE-ABS-KEY("bcd 021") OR TITLE-ABS-KEY("bcd021") OR TITLE-ABS-KEY("bevacizumab adcd") OR TITLE-ABS-KEY("bevacizumab awwb") OR TITLE-ABS-KEY("bevacizumab beta") OR TITLE-ABS-KEY("bevacizumab bvzr") OR TITLE-ABS-KEY("bevacizumab gamma") OR TITLE-ABS-KEY("bevacizumab maly") OR TITLE-ABS-KEY("bevacizumab-adcd") OR TITLE-ABS-KEY("bevacizumab-awwb") OR TITLE-ABS-KEY("bevacizumab-bvzr") OR TITLE-ABS-KEY("bevacizumab-maly") OR TITLE-ABS-KEY("bevagen") OR TITLE-ABS-KEY("bevatas") OR TITLE-ABS-KEY("bevax") OR TITLE-ABS-KEY("bevz 92") OR TITLE-ABS-KEY("bevz92") OR TITLE-ABS-KEY("bi 695502") OR TITLE-ABS-KEY("bi695502") OR TITLE-ABS-KEY("bow 030") OR TITLE-ABS-KEY("bow030") OR TITLE-ABS-KEY("boyounuo") OR TITLE-ABS-KEY("bp 01") OR TITLE-ABS-KEY("bp 102") OR TITLE-ABS-KEY("bp01") OR TITLE-ABS-KEY("bp102") OR TITLE-ABS-KEY("bryxta") OR TITLE-ABS-KEY("bs 503a") OR TITLE-ABS-KEY("bs503a") OR TITLE-ABS-KEY("bxt 2316") OR TITLE-ABS-KEY("bxt2316") OR TITLE-ABS-KEY("byvasda") OR TITLE-ABS-KEY("cbt 124") OR TITLE-ABS-KEY("cbt124") OR TITLE-ABS-KEY("chs 305") OR TITLE-ABS-KEY("chs 5217") OR TITLE-ABS-KEY("chs305") OR TITLE-ABS-KEY("chs5217") OR TITLE-ABS-KEY("cizumab") OR TITLE-ABS-KEY("ct p16") OR TITLE-ABS-KEY("ctp16") OR TITLE-ABS-KEY("equidacent") OR TITLE-ABS-KEY("fkb 238") OR TITLE-ABS-KEY("fkb238") OR TITLE-ABS-KEY("gb 222") OR TITLE-ABS-KEY("gb222") OR TITLE-ABS-KEY("gbs 004") OR TITLE-ABS-KEY("gbs004") OR TITLE-ABS-KEY("hanbeitai") OR TITLE-ABS-KEY("hd 204") OR TITLE-ABS-KEY("hd204") OR TITLE-ABS-KEY("hlx 04") OR TITLE-ABS-KEY("hlx04") OR TITLE-ABS-KEY("hot 1010") OR TITLE-ABS-KEY("hot1010") OR TITLE-ABS-KEY("ibi 305") OR TITLE-ABS-KEY("ibi305") OR TITLE-ABS-KEY("idb 0072") OR TITLE-ABS-KEY("idb0072") OR TITLE-ABS-KEY("intp 24") OR TITLE-ABS-KEY("intp24") OR TITLE-ABS-KEY("ipique") OR TITLE-ABS-KEY("jhl 1149") OR TITLE-ABS-KEY("jhl1149") OR TITLE-ABS-KEY("js 501") OR TITLE-ABS-KEY("js501") OR TITLE-ABS-KEY("jy 028") OR TITLE-ABS-KEY("jy028") OR TITLE-ABS-KEY("krabeva") OR TITLE-ABS-KEY("kyomarc") OR TITLE-ABS-KEY("lextemy") OR TITLE-ABS-KEY("lumiere (drug)") OR TITLE-ABS-KEY("ly 01008") OR TITLE-ABS-KEY("ly01008") OR TITLE-ABS-KEY("mabionvegf") OR TITLE-ABS-KEY("mb 02") OR TITLE-ABS-KEY("mb02") OR TITLE-ABS-KEY("mil 60") OR TITLE-ABS-KEY("mil60") OR TITLE-ABS-KEY("mvasi") OR TITLE-ABS-KEY("myl 14020") OR TITLE-ABS-KEY("myl 1402o") OR TITLE-ABS-KEY("myl14020") OR TITLE-ABS-KEY("myl1402o") OR TITLE-ABS-KEY("nsc 704865") OR TITLE-ABS-KEY("nsc704865") OR TITLE-ABS-KEY("onbevzi") OR TITLE-ABS-KEY("ons 1045") OR TITLE-ABS-KEY("ons 5010") OR TITLE-ABS-KEY("ons1045") OR TITLE-ABS-KEY("ons5010") OR TITLE-ABS-KEY("oyavas") OR TITLE-ABS-KEY("pf 06439535") OR TITLE-ABS-KEY("pf 6439535") OR TITLE-ABS-KEY("pf06439535") OR TITLE-ABS-KEY("pf6439535") OR TITLE-ABS-KEY("pmc 901") OR TITLE-ABS-KEY("pmc901") OR TITLE-ABS-KEY("pobevcy") OR TITLE-ABS-KEY("pro 169") OR TITLE-ABS-KEY("pro169") OR TITLE-ABS-KEY("pusintin") OR TITLE-ABS-KEY("ql 1101") OR TITLE-ABS-KEY("ql1101") OR TITLE-ABS-KEY("r 435") OR TITLE-ABS-KEY("r tpr 023") OR TITLE-ABS-KEY("r435") OR TITLE-ABS-KEY("rg 435") OR TITLE-ABS-KEY("rg435") OR TITLE-ABS-KEY("rhuMAb-VEGF") OR TITLE-ABS-KEY("ro 4876646") OR TITLE-ABS-KEY("ro4876646") OR TITLE-ABS-KEY("rph 001") OR TITLE-ABS-KEY("rph001") OR TITLE-ABS-KEY("rtpr023") OR TITLE-ABS-KEY("sb 8") OR TITLE-ABS-KEY("sb8") OR TITLE-ABS-KEY("sct 501") OR TITLE-ABS-KEY("sct 510") OR TITLE-ABS-KEY("sct501") OR TITLE-ABS-KEY("sct510") OR TITLE-ABS-KEY("stc 103") OR TITLE-ABS-KEY("stc103") OR TITLE-ABS-KEY("stivant") OR TITLE-ABS-KEY("tab 008") OR TITLE-ABS-KEY("tab 014") OR TITLE-ABS-KEY("tab008") OR TITLE-ABS-KEY("tab014") OR TITLE-ABS-KEY("tot 102") OR TITLE-ABS-KEY("tot102") OR TITLE-ABS-KEY("trs 003") OR TITLE-ABS-KEY("trs003") OR TITLE-ABS-KEY("tx 16") OR TITLE-ABS-KEY("tx16") OR TITLE-ABS-KEY("vegzelma") OR TITLE-ABS-KEY("versavo") OR TITLE-ABS-KEY("zirabev") OR TITLE-ABS-KEY("zrc 113") OR TITLE-ABS-KEY("zrc113") OR TITLE-ABS-KEY("zybev") OR TITLE-ABS-KEY("bevacizumab")) AND (TITLE-ABS-KEY("lonsurf") OR TITLE-ABS-KEY("tas 102") OR TITLE-ABS-KEY("tas102") OR TITLE-ABS-KEY("tipiracil hydrochloride plus trifluridine") OR TITLE-ABS-KEY("tipiracil hydrochloride/trifluridine") OR TITLE-ABS-KEY("tipiracil/trifluridine") OR TITLE-ABS-KEY("trifluridine plus tipiracil") OR TITLE-ABS-KEY("trifluridine plus tipiracil hydrochloride") OR TITLE-ABS-KEY("trifluridine/tipiracil") OR TITLE-ABS-KEY("trifluridine/tipiracil hydrochloride") OR TITLE-ABS-KEY("tipiracil plus trifluridine")) |
| **Web of Science  224 results** | (TS=("cancer of colon and rectum") OR TS=("cancer of rectum and colon") OR TS=("cancer of the colon and rectum") OR TS=("cancer of the colon and the rectum") OR TS=("cancer of the rectum and colon") OR TS=("cancer of the rectum and the colon") OR TS=("colo-rectal cancer") OR TS=("colo-rectal carcinogenesis") OR TS=("colo-rectal malignancies") OR TS=("colo-rectal malignancy") OR TS=("colorectal cancerogenesis") OR TS=("colorectal carcinogenesis") OR TS=("colorectal malignancies") OR TS=("colorectal malignancy") OR TS=("malignancies of the colon and rectum") OR TS=("malignancy of colon and rectum") OR TS=("malignancy of the colon and rectum") OR TS=("recto-colonic cancer") OR TS=("rectocolonic cancer") OR TS=("colorectal cancer") OR TS=("colorectal cancers") OR TS=("Colorectal Neoplasm") OR TS=("Colorectal Tumors") OR TS=("Colorectal Tumor") OR TS=("colorectal carcinoma") OR TS=("colorectal carcinomas")) AND (TS=("abevmy") OR TS=("abp 215") OR TS=("abp215") OR TS=("ainex") OR TS=("altuzan") OR TS=("alymsys") OR TS=("ankeda") OR TS=("ask b1202") OR TS=("askb1202") OR TS=("avastin") OR TS=("avegra") OR TS=("aybintio") OR TS=("ba 1101") OR TS=("ba1101") OR TS=("bambevi") OR TS=("bat 1706") OR TS=("bat1706") OR TS=("bcd 021") OR TS=("bcd021") OR TS=("bevacizumab adcd") OR TS=("bevacizumab awwb") OR TS=("bevacizumab beta") OR TS=("bevacizumab bvzr") OR TS=("bevacizumab gamma") OR TS=("bevacizumab maly") OR TS=("bevacizumab-adcd") OR TS=("bevacizumab-awwb") OR TS=("bevacizumab-bvzr") OR TS=("bevacizumab-maly") OR TS=("bevagen") OR TS=("bevatas") OR TS=("bevax") OR TS=("bevz 92") OR TS=("bevz92") OR TS=("bi 695502") OR TS=("bi695502") OR TS=("bow 030") OR TS=("bow030") OR TS=("boyounuo") OR TS=("bp 01") OR TS=("bp 102") OR TS=("bp01") OR TS=("bp102") OR TS=("bryxta") OR TS=("bs 503a") OR TS=("bs503a") OR TS=("bxt 2316") OR TS=("bxt2316") OR TS=("byvasda") OR TS=("cbt 124") OR TS=("cbt124") OR TS=("chs 305") OR TS=("chs 5217") OR TS=("chs305") OR TS=("chs5217") OR TS=("cizumab") OR TS=("ct p16") OR TS=("ctp16") OR TS=("equidacent") OR TS=("fkb 238") OR TS=("fkb238") OR TS=("gb 222") OR TS=("gb222") OR TS=("gbs 004") OR TS=("gbs004") OR TS=("hanbeitai") OR TS=("hd 204") OR TS=("hd204") OR TS=("hlx 04") OR TS=("hlx04") OR TS=("hot 1010") OR TS=("hot1010") OR TS=("ibi 305") OR TS=("ibi305") OR TS=("idb 0072") OR TS=("idb0072") OR TS=("intp 24") OR TS=("intp24") OR TS=("ipique") OR TS=("jhl 1149") OR TS=("jhl1149") OR TS=("js 501") OR TS=("js501") OR TS=("jy 028") OR TS=("jy028") OR TS=("krabeva") OR TS=("kyomarc") OR TS=("lextemy") OR TS=("lumiere (drug)") OR TS=("ly 01008") OR TS=("ly01008") OR TS=("mabionvegf") OR TS=("mb 02") OR TS=("mb02") OR TS=("mil 60") OR TS=("mil60") OR TS=("mvasi") OR TS=("myl 14020") OR TS=("myl 1402o") OR TS=("myl14020") OR TS=("myl1402o") OR TS=("nsc 704865") OR TS=("nsc704865") OR TS=("onbevzi") OR TS=("ons 1045") OR TS=("ons 5010") OR TS=("ons1045") OR TS=("ons5010") OR TS=("oyavas") OR TS=("pf 06439535") OR TS=("pf 6439535") OR TS=("pf06439535") OR TS=("pf6439535") OR TS=("pmc 901") OR TS=("pmc901") OR TS=("pobevcy") OR TS=("pro 169") OR TS=("pro169") OR TS=("pusintin") OR TS=("ql 1101") OR TS=("ql1101") OR TS=("r 435") OR TS=("r tpr 023") OR TS=("r435") OR TS=("rg 435") OR TS=("rg435") OR TS=("rhuMAb-VEGF") OR TS=("ro 4876646") OR TS=("ro4876646") OR TS=("rph 001") OR TS=("rph001") OR TS=("rtpr023") OR TS=("sb 8") OR TS=("sb8") OR TS=("sct 501") OR TS=("sct 510") OR TS=("sct501") OR TS=("sct510") OR TS=("stc 103") OR TS=("stc103") OR TS=("stivant") OR TS=("tab 008") OR TS=("tab 014") OR TS=("tab008") OR TS=("tab014") OR TS=("tot 102") OR TS=("tot102") OR TS=("trs 003") OR TS=("trs003") OR TS=("tx 16") OR TS=("tx16") OR TS=("vegzelma") OR TS=("versavo") OR TS=("zirabev") OR TS=("zrc 113") OR TS=("zrc113") OR TS=("zybev") OR TS=("bevacizumab")) AND (TS=("lonsurf") OR TS=("tas 102") OR TS=("tas102") OR TS=("tipiracil hydrochloride plus trifluridine") OR TS=("tipiracil hydrochloride/trifluridine") OR TS=("tipiracil/trifluridine") OR TS=("trifluridine plus tipiracil") OR TS=("trifluridine plus tipiracil hydrochloride") OR TS=("trifluridine/tipiracil") OR TS=("trifluridine/tipiracil hydrochloride") OR TS=("tipiracil plus trifluridine")) |
| **The Cochrane Library  97 results** | (("cancer of colon and rectum"):ti,ab,kw OR ("cancer of rectum and colon"):ti,ab,kw OR ("cancer of the colon and rectum"):ti,ab,kw OR ("cancer of the colon and the rectum"):ti,ab,kw OR ("cancer of the rectum and colon"):ti,ab,kw OR ("cancer of the rectum and the colon"):ti,ab,kw OR ("colo-rectal cancer"):ti,ab,kw OR ("colo-rectal carcinogenesis"):ti,ab,kw OR ("colo-rectal malignancies"):ti,ab,kw OR ("colo-rectal malignancy"):ti,ab,kw OR ("colorectal cancerogenesis"):ti,ab,kw OR ("colorectal carcinogenesis"):ti,ab,kw OR ("colorectal malignancies"):ti,ab,kw OR ("colorectal malignancy"):ti,ab,kw OR ("malignancies of the colon and rectum"):ti,ab,kw OR ("malignancy of colon and rectum"):ti,ab,kw OR ("malignancy of the colon and rectum"):ti,ab,kw OR ("recto-colonic cancer"):ti,ab,kw OR ("rectocolonic cancer"):ti,ab,kw OR ("colorectal cancer"):ti,ab,kw OR ("colorectal cancers"):ti,ab,kw OR ("Colorectal Neoplasm"):ti,ab,kw OR ("Colorectal Tumors"):ti,ab,kw OR ("Colorectal Tumor"):ti,ab,kw OR ("colorectal carcinoma"):ti,ab,kw OR ("colorectal carcinomas"):ti,ab,kw) AND (("abevmy"):ti,ab,kw OR ("abp 215"):ti,ab,kw OR ("abp215"):ti,ab,kw OR ("ainex"):ti,ab,kw OR ("altuzan"):ti,ab,kw OR ("alymsys"):ti,ab,kw OR ("ankeda"):ti,ab,kw OR ("ask b1202"):ti,ab,kw OR ("askb1202"):ti,ab,kw OR ("avastin"):ti,ab,kw OR ("avegra"):ti,ab,kw OR ("aybintio"):ti,ab,kw OR ("ba 1101"):ti,ab,kw OR ("ba1101"):ti,ab,kw OR ("bambevi"):ti,ab,kw OR ("bat 1706"):ti,ab,kw OR ("bat1706"):ti,ab,kw OR ("bcd 021"):ti,ab,kw OR ("bcd021"):ti,ab,kw OR ("bevacizumab adcd"):ti,ab,kw OR ("bevacizumab awwb"):ti,ab,kw OR ("bevacizumab beta"):ti,ab,kw OR ("bevacizumab bvzr"):ti,ab,kw OR ("bevacizumab gamma"):ti,ab,kw OR ("bevacizumab maly"):ti,ab,kw OR ("bevacizumab-adcd"):ti,ab,kw OR ("bevacizumab-awwb"):ti,ab,kw OR ("bevacizumab-bvzr"):ti,ab,kw OR ("bevacizumab-maly"):ti,ab,kw OR ("bevagen"):ti,ab,kw OR ("bevatas"):ti,ab,kw OR ("bevax"):ti,ab,kw OR ("bevz 92"):ti,ab,kw OR ("bevz92"):ti,ab,kw OR ("bi 695502"):ti,ab,kw OR ("bi695502"):ti,ab,kw OR ("bow 030"):ti,ab,kw OR ("bow030"):ti,ab,kw OR ("boyounuo"):ti,ab,kw OR ("bp 01"):ti,ab,kw OR ("bp 102"):ti,ab,kw OR ("bp01"):ti,ab,kw OR ("bp102"):ti,ab,kw OR ("bryxta"):ti,ab,kw OR ("bs 503a"):ti,ab,kw OR ("bs503a"):ti,ab,kw OR ("bxt 2316"):ti,ab,kw OR ("bxt2316"):ti,ab,kw OR ("byvasda"):ti,ab,kw OR ("cbt 124"):ti,ab,kw OR ("cbt124"):ti,ab,kw OR ("chs 305"):ti,ab,kw OR ("chs 5217"):ti,ab,kw OR ("chs305"):ti,ab,kw OR ("chs5217"):ti,ab,kw OR ("cizumab"):ti,ab,kw OR ("ct p16"):ti,ab,kw OR ("ctp16"):ti,ab,kw OR ("equidacent"):ti,ab,kw OR ("fkb 238"):ti,ab,kw OR ("fkb238"):ti,ab,kw OR ("gb 222"):ti,ab,kw OR ("gb222"):ti,ab,kw OR ("gbs 004"):ti,ab,kw OR ("gbs004"):ti,ab,kw OR ("hanbeitai"):ti,ab,kw OR ("hd 204"):ti,ab,kw OR ("hd204"):ti,ab,kw OR ("hlx 04"):ti,ab,kw OR ("hlx04"):ti,ab,kw OR ("hot 1010"):ti,ab,kw OR ("hot1010"):ti,ab,kw OR ("ibi 305"):ti,ab,kw OR ("ibi305"):ti,ab,kw OR ("idb 0072"):ti,ab,kw OR ("idb0072"):ti,ab,kw OR ("intp 24"):ti,ab,kw OR ("intp24"):ti,ab,kw OR ("ipique"):ti,ab,kw OR ("jhl 1149"):ti,ab,kw OR ("jhl1149"):ti,ab,kw OR ("js 501"):ti,ab,kw OR ("js501"):ti,ab,kw OR ("jy 028"):ti,ab,kw OR ("jy028"):ti,ab,kw OR ("krabeva"):ti,ab,kw OR ("kyomarc"):ti,ab,kw OR ("lextemy"):ti,ab,kw OR ("lumiere (drug)"):ti,ab,kw OR ("ly 01008"):ti,ab,kw OR ("ly01008"):ti,ab,kw OR ("mabionvegf"):ti,ab,kw OR ("mb 02"):ti,ab,kw OR ("mb02"):ti,ab,kw OR ("mil 60"):ti,ab,kw OR ("mil60"):ti,ab,kw OR ("mvasi"):ti,ab,kw OR ("myl 14020"):ti,ab,kw OR ("myl 1402o"):ti,ab,kw OR ("myl14020"):ti,ab,kw OR ("myl1402o"):ti,ab,kw OR ("nsc 704865"):ti,ab,kw OR ("nsc704865"):ti,ab,kw OR ("onbevzi"):ti,ab,kw OR ("ons 1045"):ti,ab,kw OR ("ons 5010"):ti,ab,kw OR ("ons1045"):ti,ab,kw OR ("ons5010"):ti,ab,kw OR ("oyavas"):ti,ab,kw OR ("pf 06439535"):ti,ab,kw OR ("pf 6439535"):ti,ab,kw OR ("pf06439535"):ti,ab,kw OR ("pf6439535"):ti,ab,kw OR ("pmc 901"):ti,ab,kw OR ("pmc901"):ti,ab,kw OR ("pobevcy"):ti,ab,kw OR ("pro 169"):ti,ab,kw OR ("pro169"):ti,ab,kw OR ("pusintin"):ti,ab,kw OR ("ql 1101"):ti,ab,kw OR ("ql1101"):ti,ab,kw OR ("r 435"):ti,ab,kw OR ("r tpr 023"):ti,ab,kw OR ("r435"):ti,ab,kw OR ("rg 435"):ti,ab,kw OR ("rg435"):ti,ab,kw OR ("rhuMAb-VEGF"):ti,ab,kw OR ("ro 4876646"):ti,ab,kw OR ("ro4876646"):ti,ab,kw OR ("rph 001"):ti,ab,kw OR ("rph001"):ti,ab,kw OR ("rtpr023"):ti,ab,kw OR ("sb 8"):ti,ab,kw OR ("sb8"):ti,ab,kw OR ("sct 501"):ti,ab,kw OR ("sct 510"):ti,ab,kw OR ("sct501"):ti,ab,kw OR ("sct510"):ti,ab,kw OR ("stc 103"):ti,ab,kw OR ("stc103"):ti,ab,kw OR ("stivant"):ti,ab,kw OR ("tab 008"):ti,ab,kw OR ("tab 014"):ti,ab,kw OR ("tab008"):ti,ab,kw OR ("tab014"):ti,ab,kw OR ("tot 102"):ti,ab,kw OR ("tot102"):ti,ab,kw OR ("trs 003"):ti,ab,kw OR ("trs003"):ti,ab,kw OR ("tx 16"):ti,ab,kw OR ("tx16"):ti,ab,kw OR ("vegzelma"):ti,ab,kw OR ("versavo"):ti,ab,kw OR ("zirabev"):ti,ab,kw OR ("zrc 113"):ti,ab,kw OR ("zrc113"):ti,ab,kw OR ("zybev"):ti,ab,kw OR ("bevacizumab"):ti,ab,kw) AND (("lonsurf"):ti,ab,kw OR ("tas 102"):ti,ab,kw OR ("tas102"):ti,ab,kw OR ("tipiracil hydrochloride plus trifluridine"):ti,ab,kw OR ("tipiracil hydrochloride/trifluridine"):ti,ab,kw OR ("tipiracil/trifluridine"):ti,ab,kw OR ("trifluridine plus tipiracil"):ti,ab,kw OR ("trifluridine plus tipiracil hydrochloride"):ti,ab,kw OR ("trifluridine/tipiracil"):ti,ab,kw OR ("trifluridine/tipiracil hydrochloride"):ti,ab,kw OR ("tipiracil plus trifluridine"):ti,ab,kw) |
|  |  |

**Table S5** Treatment regimens from trials included in this systematic review and meta-analysis

| **Study** | **Treatment regimes** |
| --- | --- |
| CHIDA et al., 2021 | Non specified. |
| FUJII et al., 2019 | Patients were treated with TAS-102 (35 mg/m² of body surface area) orally twice a day on days 1–5 and 8–12 in a 28-day cycle with or without Bmab (5 mg/kg of bodyweight, administered by intravenous infusion for 30 minutes every 2 weeks). Patients were all administered an initial regular dose of chemotherapy in the ﬁrst cycle. Dose reduction was performed in the subsequent chemotherapy cycle for patients who suffered severe adverse events, particularly grade 3–4 neutropenia. In these patients, the dose of TAS-102 was reduced by 10 mg/day when necessary on a course basis, and no dose escalation was performed, even if the adverse events disappeared. Treatment was delayed when one or more of the following adverse events occurred: grade 3–4 neutropenia or thrombocytopenia, febrile neutropenia, total bilirubin >3.0 mg/dL, aspartate transaminase and alanine transaminase >150 U/L, creatinine >1.5 mg/dL, and grade 3–4 nonhematological toxicity. Treatment was restarted after recovery from the severe adverse events by reducing the dose of TAS-102 by 10 mg/day. |
| KOTANI et al., 2019 | Trifluridine/tipiracil plus bevacizumab regimen consisted of trifluridine/tipiracil 35 mg/m² of body surface area, given orally twice a day on days 1–5 and 8–12 in a 28-day cycle, and bevacizumab 5 mg/kg of bodyweight, administered by intravenous infusion every 2 weeks. Trifluridine/tipiracil monotherapy consisted of trifluridine/tipiracil 35 mg/m² of body surface area, given orally twice a day on days 1–5 and 8–12 in a 28-day cycle. |
| NIE et al., 2023 | In this study, patients received TAS-102 as third-line or above therapy in 3 treatment modalities, including TAS-102 monotherapy, TAS-102 in combination with bevacizumab and TAS-102 combined with ICIs. In TAS-102 monotherapy group, TAS-102 was administered orally with 4 weeks as 1 treatment cycle, and the dosage was 35 mg per square meter twice daily from d1-d5 in the ﬁrst week and d8-d12 in the second week, followed by a 2-weeks rest period. In TAS-102 plus bevacizumab group, TAS-102 was administered at the same dose and administration mode. Bevacizumab was administered by intravenous, and the dosage was 5 mg/kg once every 2 weeks. |
| PFEIFFER et al., 2020 | Patients assigned to monotherapy were treated with TAS­102 35 mg/m² orally twice daily on days 1–5 and 8–12 every 28 days. Patients assigned to combination therapy received TAS­102 plus bevacizumab (5 mg/kg intravenously) on days 1 and 15 every 28 days. The bevacizumab dose was administered as a 30­ min intravenous infusion before the TAS­102 dose.  If dose reduction was needed during treatment because of toxicity, the dose of TAS­102 was reduced in increments of 5 mg/m². If patients had unacceptable toxicities related to bevacizumab, treatment with TAS­102 monotherapy could be continued according to protocol without bevacizumab. Dose reduction of bevacizumab was not recommended. In the case of treatment delay of TAS­102, bevacizumab administration could be delayed as well. The protocol recommended treatment until progression, unacceptable toxicity, or patients’ wish for a treatment break. Prophylactic use of granulocyte colony­ stimulating factor was not recommended, but it was an option in cases of febrile neutropenia or delay in treatment administration because of neutropenia. |
| PRAGER et al., 2023 | Patients were randomly assigned in a 1:1 ratio to receive FTD–TPI (Lonsurf, Servier and Taiho Oncology) plus bevacizumab (Avastin, Genentech and Roche) (combination group) or FTD– TPI alone (FTD–TPI group). Randomization was stratified according to geographic region (North America, European Union, or rest of the world), time since diagnosis of first metastasis (<18 months or ≥18 months), and RAS status (wild type or mutated). FTD–TPI was administered orally, twice daily, at a starting dose of 35 mg per square meter of body-surface area, on days 1 through 5 and on days 8 through 12 every 28 days. Bevacizumab, at a dose of 5 mg per kilogram of body weight, was administered intravenously on days 1 and 15. The 28-day treatment cycle continued until disease progression or unacceptable toxic effects occurred or consent was withdrawn. Patients were considered to be receiving treatment for as long as they continued to receive FTD–TPI; bevacizumab monotherapy was not allowed. |
| SHIBUTANI et al., 2020. | Patients were treated with FTD/TPI (35 mg/m² of body surface area) orally twice a day on days 1-5 and 8-12 in a 28-day cycle with or without bevacizumab (5 mg/kg of body wight) administered by intravenous infusion every 2 weeks. |

**Table S6 -** Baseline characteristics of included studies

|  | | PFEIFFER et al., 2020 | | CHIDA et al., 2021 | | PRAGER et al., 2023 | | KOTANI et al., 2019 | | FUJII et al., 2019 | | SHIBUTANI et al., 2020 | NIE et al., 2023 | |  |
| --- | --- | --- | --- | --- | --- | --- | --- | --- | --- | --- | --- | --- | --- | --- | --- |
|  |  |  |  |  |  |  |  |  |  |  |  |  |  |  |  |
| **Region (%)** | North America | NA | | NA | | 8 (3.3) | 8 (3.3) | NA | | NA | | NA | NA | |  |
|  | European Union |  |  |  |  | 158 (64.2) | 157 (63.8) |  |  |  |  |  |  |  |  |
|  | Rest of the World |  |  |  |  | 80 (32.5) | 81 (32.9) |  |  |  |  |  |  |  |  |
| **Race (%)** | White | NA | | NA | | 215 (87.4) | 220 (89.4) | NA | | NA | | NA | NA | |  |
|  | Black |  |  |  |  | 4 (1.6) | 3 (1.2) |  |  |  |  |  |  |  |  |
|  | Asian |  |  |  |  | 0 | 1 (0.4) |  |  |  |  |  |  |  |  |
|  | American Indian or Alaska Native |  |  |  |  | 1 (0.4) | 0 |  |  |  |  |  |  |  |  |
|  | Other |  |  |  |  | 8 (3.3) | 5 (2.0) |  |  |  |  |  |  |  |  |
|  | Unknown |  |  |  |  | 18 (7.3) | 17 (6.9) |  |  |  |  |  |  |  |  |
| **Site of metastatic disease (%)** | Primary | 6 (13) | 12 (25.5) | - | - | NA | | NA | | - | - | NA | - |  |  |
|  | Liver | 28 (60.8) | 40 (85.1) | 88 (63.3) | 97 (63.4) |  |  |  |  | 15 (71) | 25 (69) |  | 33 (61.1) | 4 (66.7) |  |
|  | Lung | 31 (67.4) | 34 (72.3) | 91 (65.5) | 79 (51.6) |  |  |  |  | 15 (71) | 28 (78) |  | 28 (51.9) | 5 (83.3) |  |
|  | Lymph nodes | 18 (39.1) | 18 (38.2) | - | - |  |  |  |  | 5 (24) | 11 (31) |  | 26 (48.1) | 4 (66.7) |  |
|  | Peritoneum | 6 (13) | 8 (17) | 31 (22.3) | 35 (22.9) |  |  |  |  | 4 (19) | 12 (33) |  | 13 (24.1) | 3 (50.0) |  |
|  | Bone | 7 (15.2) | 1 (2.1) | - | - |  |  |  |  | - | - |  | Others = 21 (38.9) | Others = 3 (50.0) |  |
|  | Soft tissue | 3 (6.5) | 7 (14.8) | - | - |  |  |  |  | - | - |  | - | - |  |
| **MMR and MSI status (%)** | MMR deficient and high MSI | NA | | NA | | 13 (5.3) | 8 (3.3) | NA | | NA | | NA | - | - |  |
|  | MMR proficient and stable or low MSI |  |  |  |  | 139 (56.5) | 145 (58.9) |  |  |  |  |  | 48 (88.9) | 6 (100.0) |  |
|  | Unknown or missing data |  |  |  |  | 94 (38.2) | 93 (37.8) |  |  |  |  |  | 6 (11.1) | 0 (0) |  |
| **Previous lines of therapy (%)** | ≤2 | 21 (45.6) | 20 (42.5) | NA | | 240 (97.6) | 239 (97.2) | 30 (50) | 35 (53) | NA | | NA | 25 (46.3) | 2 (33.3) |  |
|  | 3 | 12 (26.1) | 13 (27.7) |  |  | 6 (2.4) | 7 (2.8) | 15 (25.0) | 16 (24.2) |  |  |  | 29 (53.7) | 4 (66.7) |  |
|  | 4 | 8 (17.4) | 8 (17) |  |  |  |  | 12 (20.0) | 15 (22.7) |  |  |  |  |  |  |
|  | ≥5 | 5 (10.9) | 6 (12.8) |  |  |  |  |  |  |  |  |  |  |  |  |
| **Previous therapy for metastatic colorectal cancer (%)** | Fluoropyrimidines | 46 (100) | 47 (100) | 139 (100.0) | 153 (100.0) | 246 (100.0) | 246 (100.0) | 60 (100) | 66 (100) | NA | | NA | 54 (100.0) | 6 (100.0) |  |
|  | Oxaliplatin | 45 (97.8) | 47 (100) | 139 (100.0) | 153 (100.0) | 241 (98.0) | 243 (98.8) | 60 (100) | 66 (100) |  |  |  | 54 (100.0) | 6 (100.0) |  |
|  | Irinotecan | 46 (100) | 47 (100) | 139 (100.0) | 153 (100.0) | 246 (100.0) | 245 (99.6) | 60 (100) | 66 (100) |  |  |  | 47 (87.0) | 6 (100.0) |  |
|  | Anti-VEGF therapy | 39 (91.3) | 39 (83) | 127 (91.0) | 139 (90.8) | 178 (72.4) | 176 (71.5) | 58 (96.7) | 61 (92.4) |  |  |  | 50 (92.6) | 6 (100.0) |  |
|  | Anti- EGFR therapy | 19 (41.3) | 18 (38.3) | 56 (100.0) | 48 (100.0) | 67 (27.2) | 66 (26.8) | 27 (45.0) | 27 (40.9) |  |  |  | 15 (27.8) | 1 (16.7) |  |
| **Neutrophils ≥5 × 10⁹ cells per L** | | 21 (46%) | 23 (49%) | NA | | NA | | NA | | NA | | NA | NA | |  |
| **Neutrophil–lymphocyte ratio — no./total no. (%)** | <3 | NA | | NA | | 128/245 (52.0) | 115/246 (46.7) | NA | | NA | | NA | NA | |  |
|  | ≥3 |  |  |  |  | 117/245 (47.6) | 131/246 (53.3) |  |  |  |  |  |  |  |  |

No – Number; MMR – Mismatch repair; MSI - Microsatellite Instability.

**Supplementary Figure 1** Any grade of adverse events. **A.** Neutropenia. **B.** Thrombocytopenia. **C.** Nausea. **D.** Diarrhea**. E.** Vomiting. **F.** Fatigue**. G.** Febrile neutropenia. **H.** Anaemia. Comparison between Trifluridine-tipiracil plus Bevacizumab versus Trifluridine-tipiracil monotherapy in patients with metastatic colorectal cancer.

**A. Neutropenia**

**
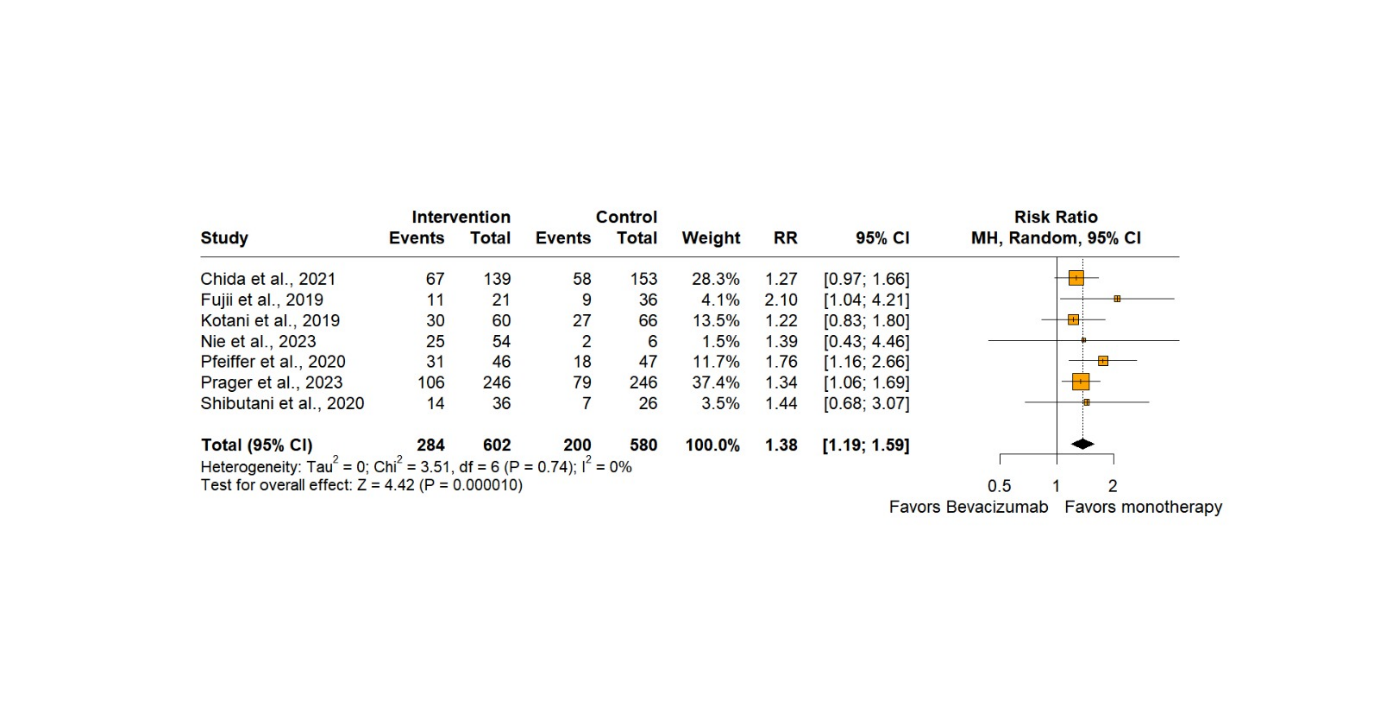
**

**B. Thrombocytopenia**

**
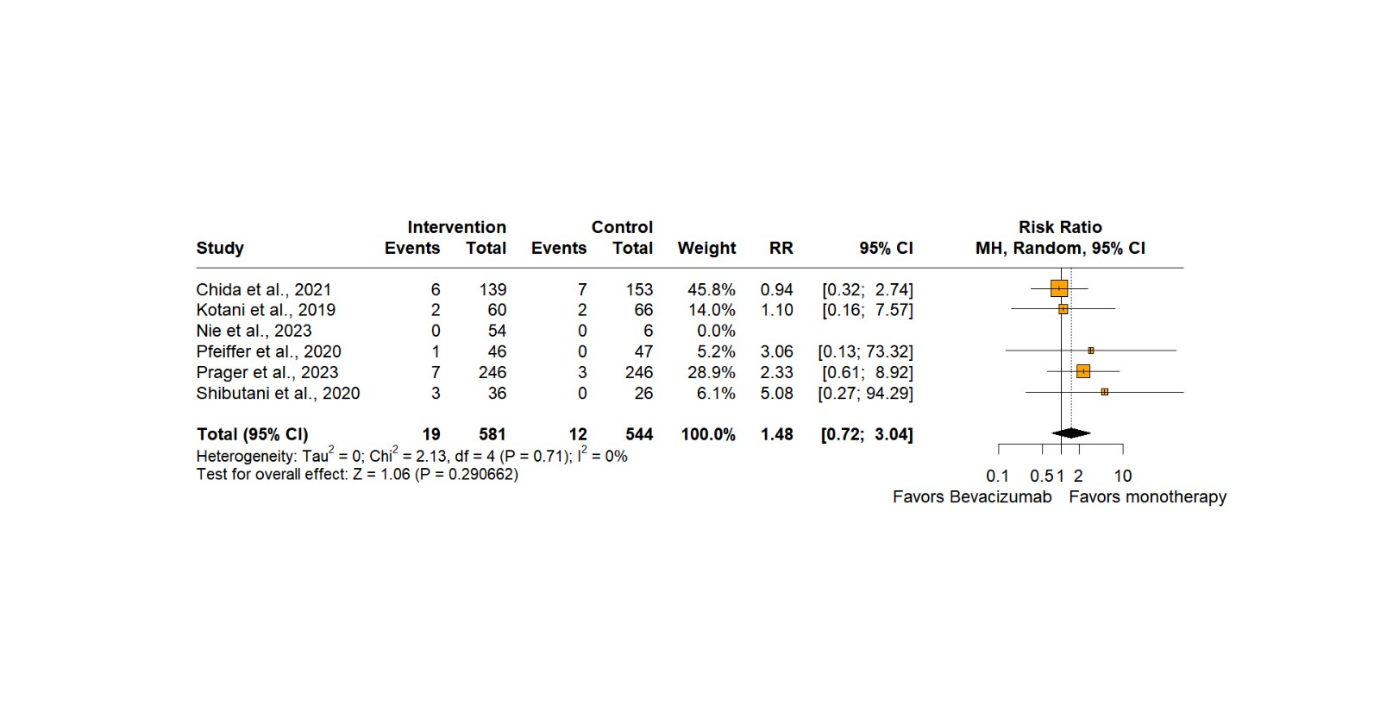
**

**C. Nausea**

**
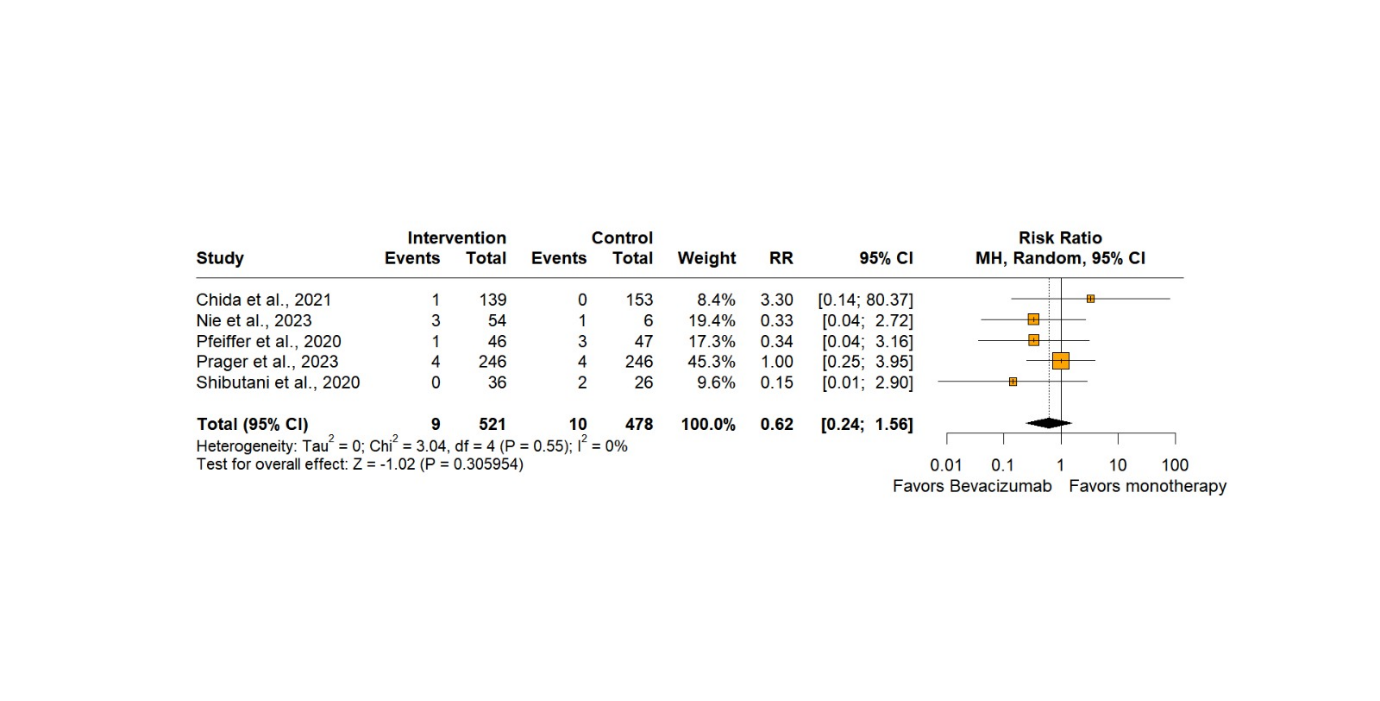
**

**D. Diarrhea**

**
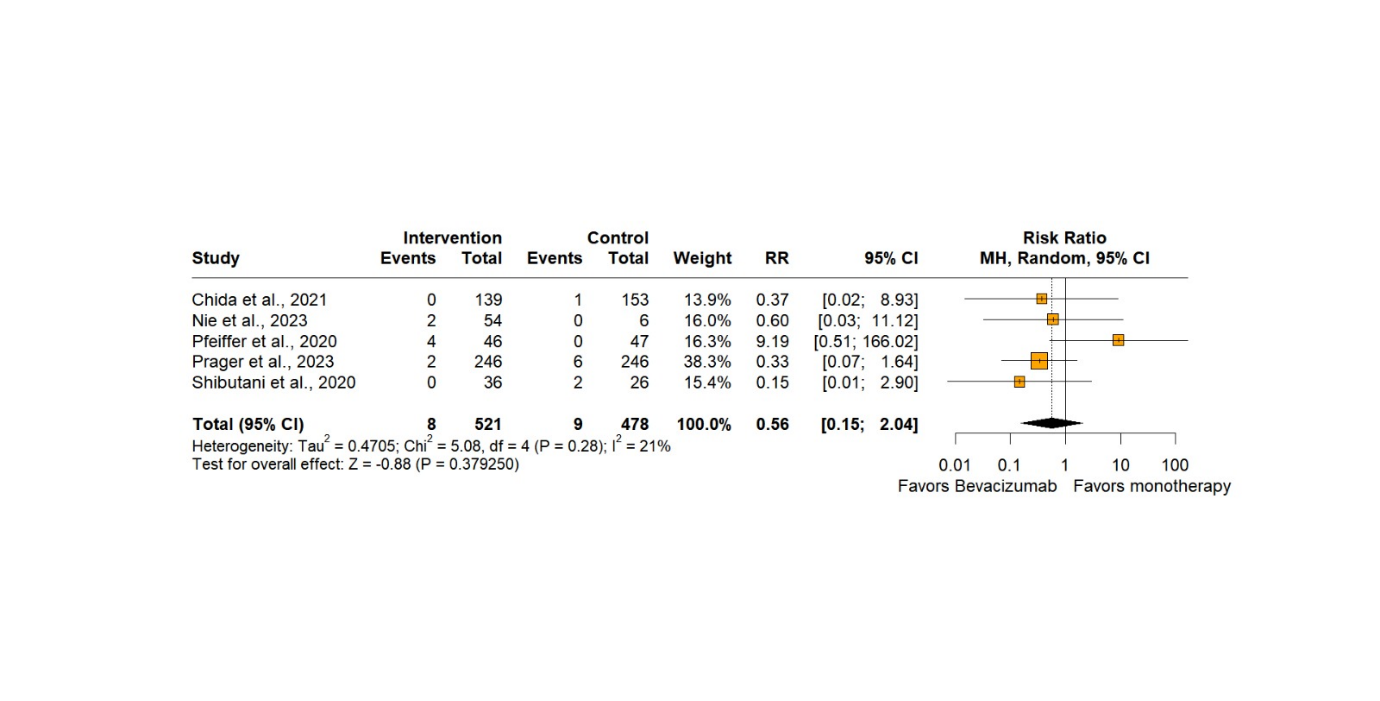
**

**E. Vomiting**

**
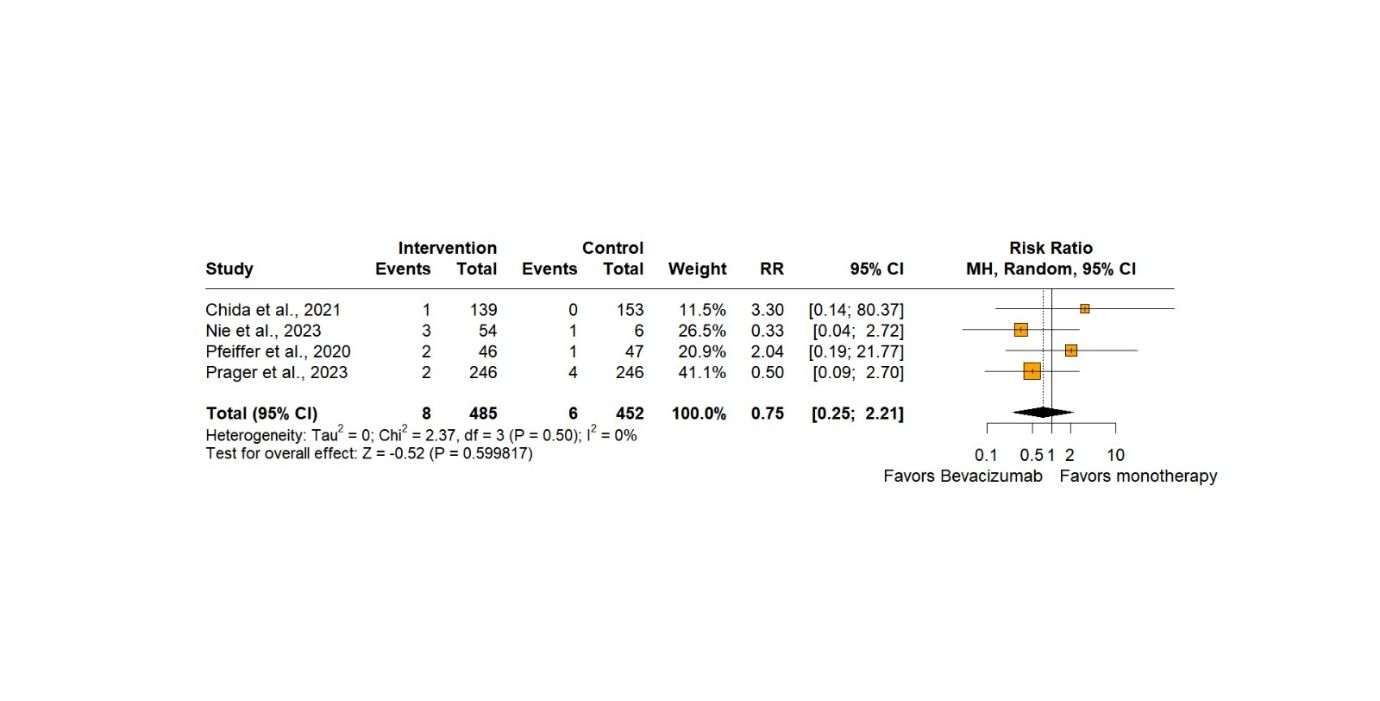
**

**F. Fatigue**

**
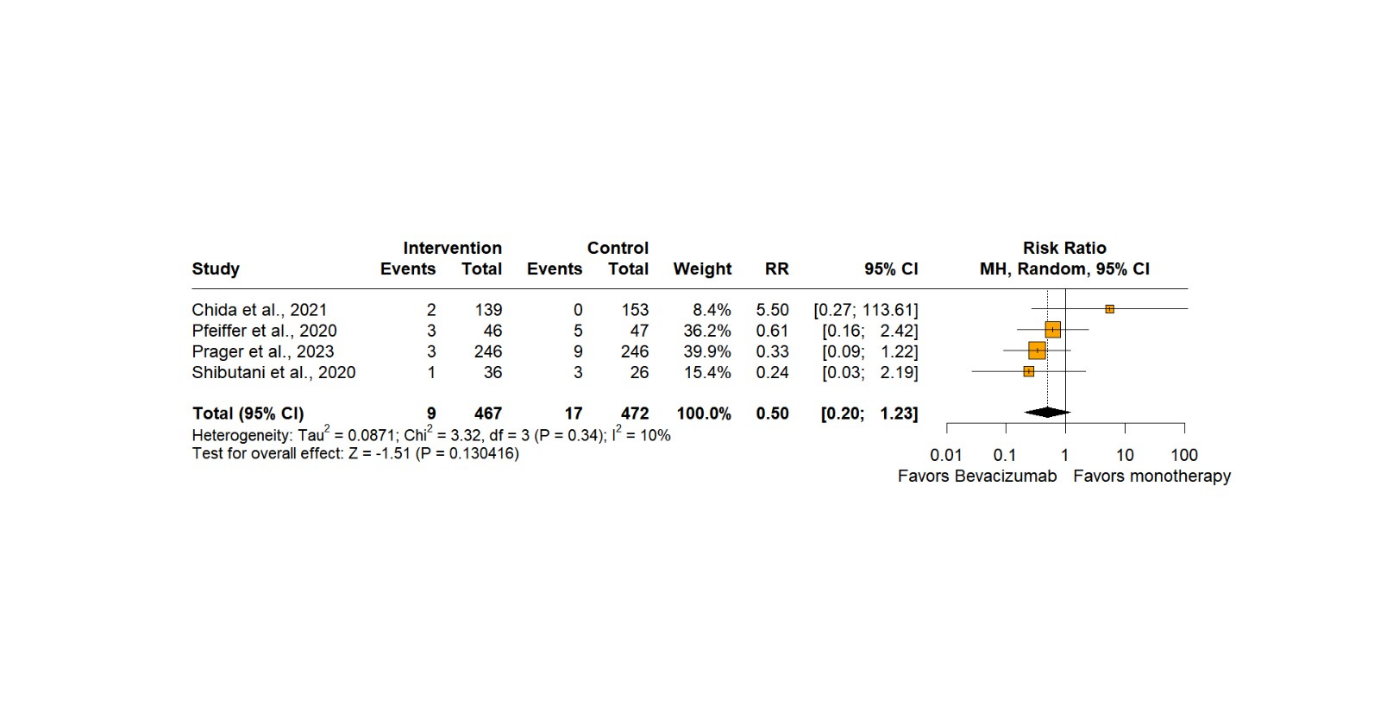
**

**G. Febrile Neutropenia**

**
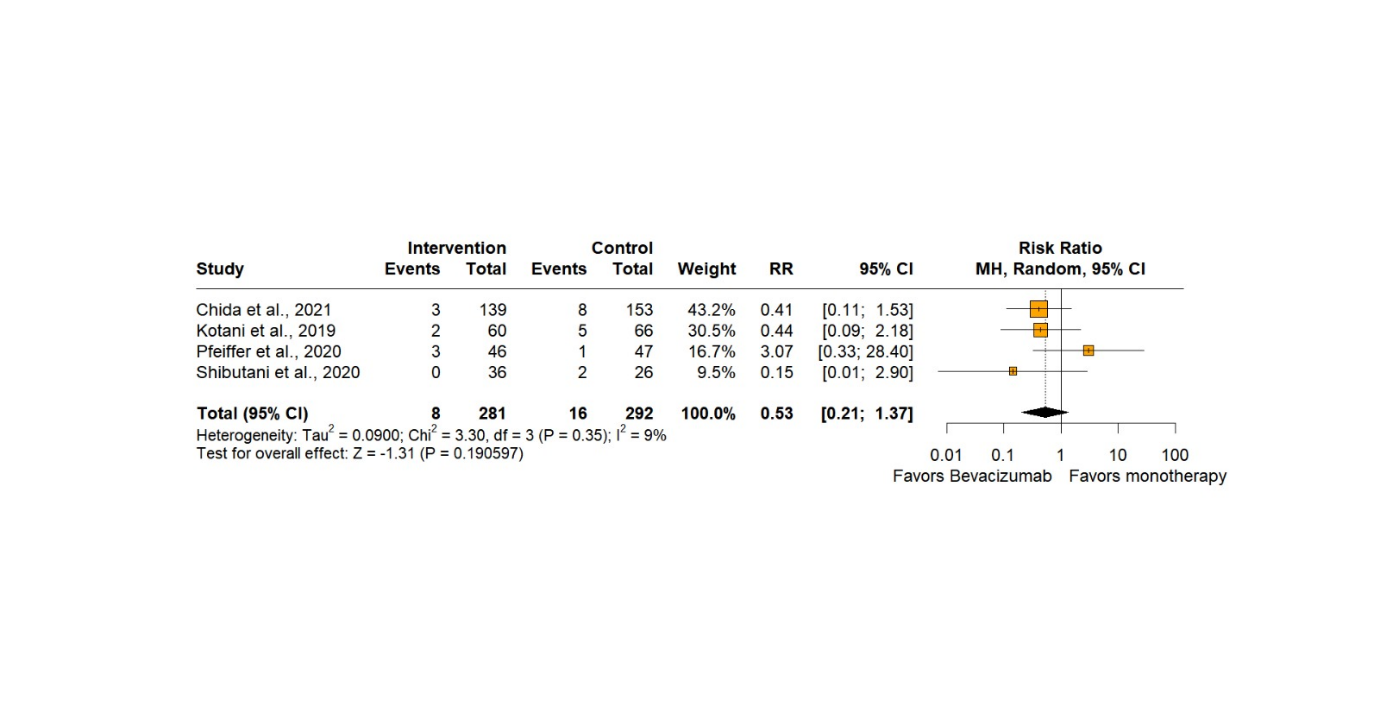
**

**H. Anaemia**

**
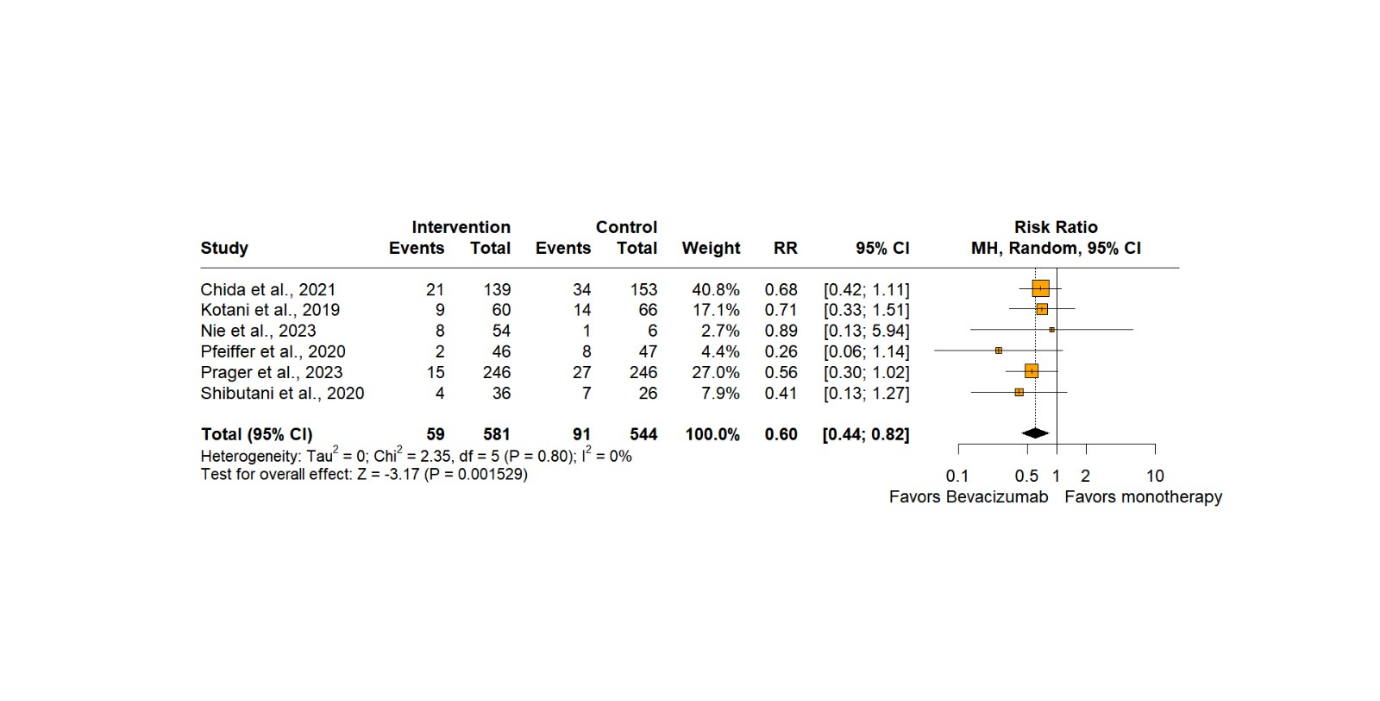
**

**Supplementary Figure 2** Leave-one-out sensitivity analyses. **A.** Progression-free survival overall **B.** Overall survival. **C.** Objective response rate (ORR). **D.** Disease Control Rate (DCR).

**A. Progression-free survival overall**


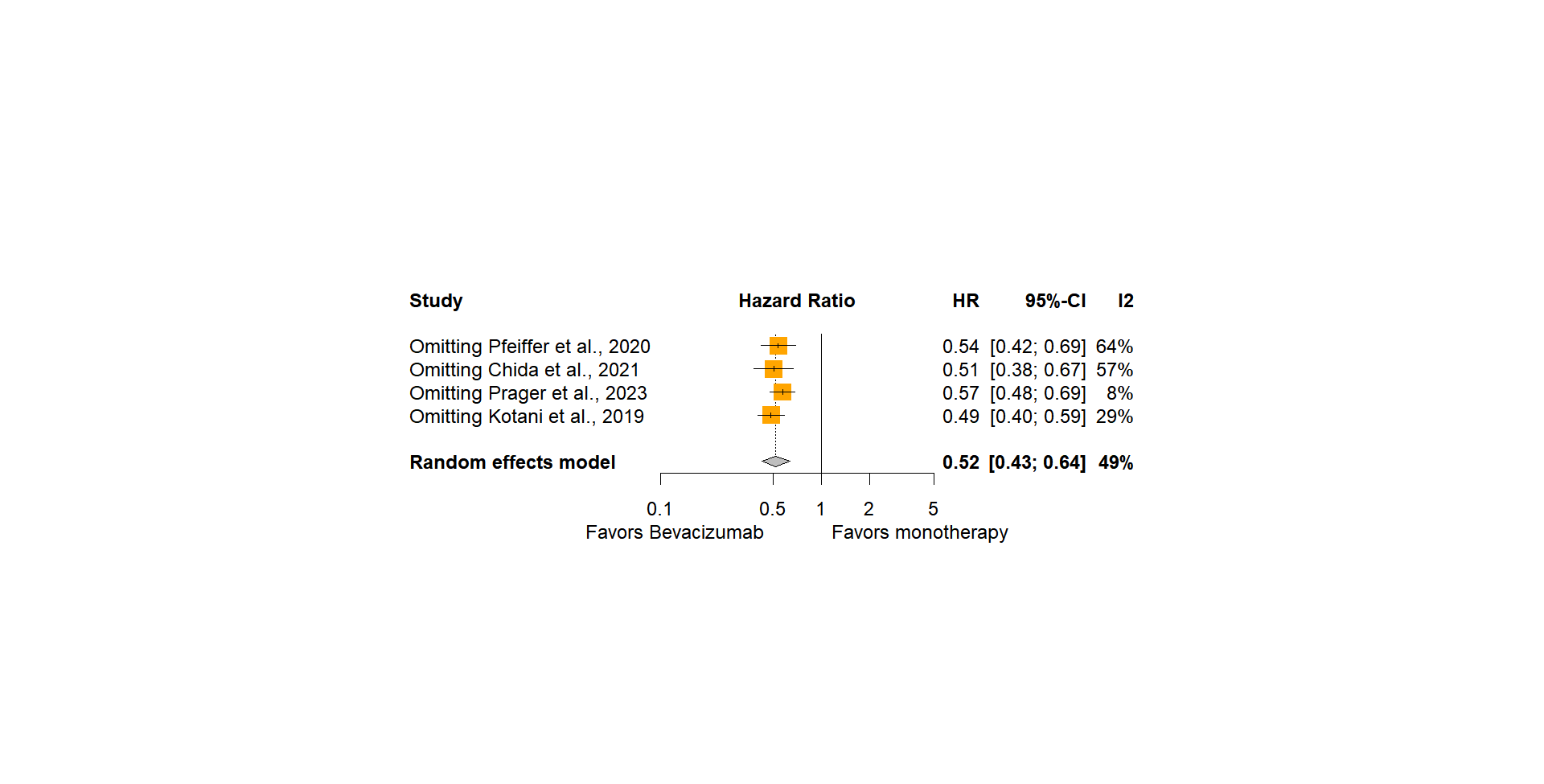


**B. Overall survival**


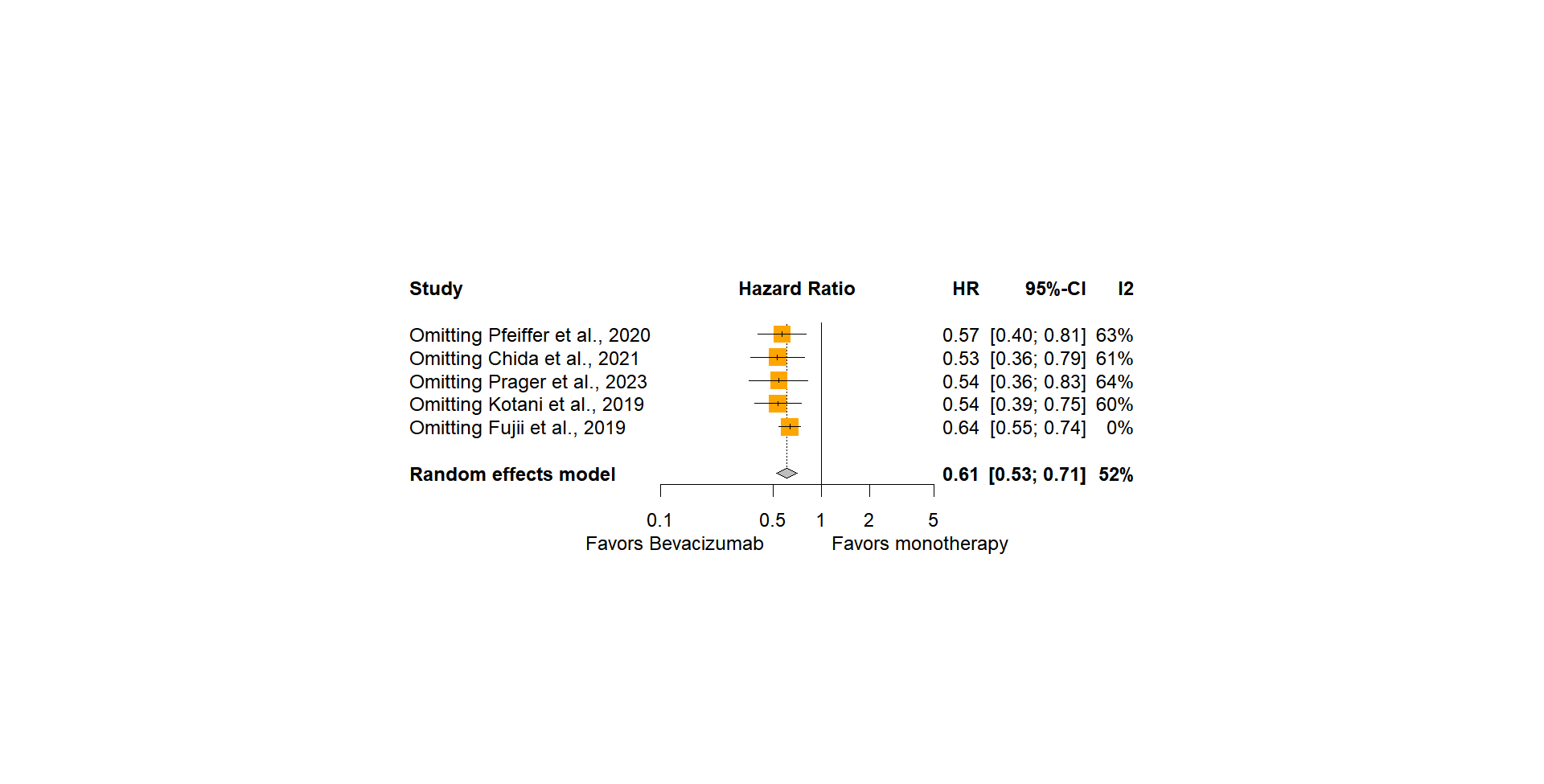


**C. Objective response rate (ORR)**


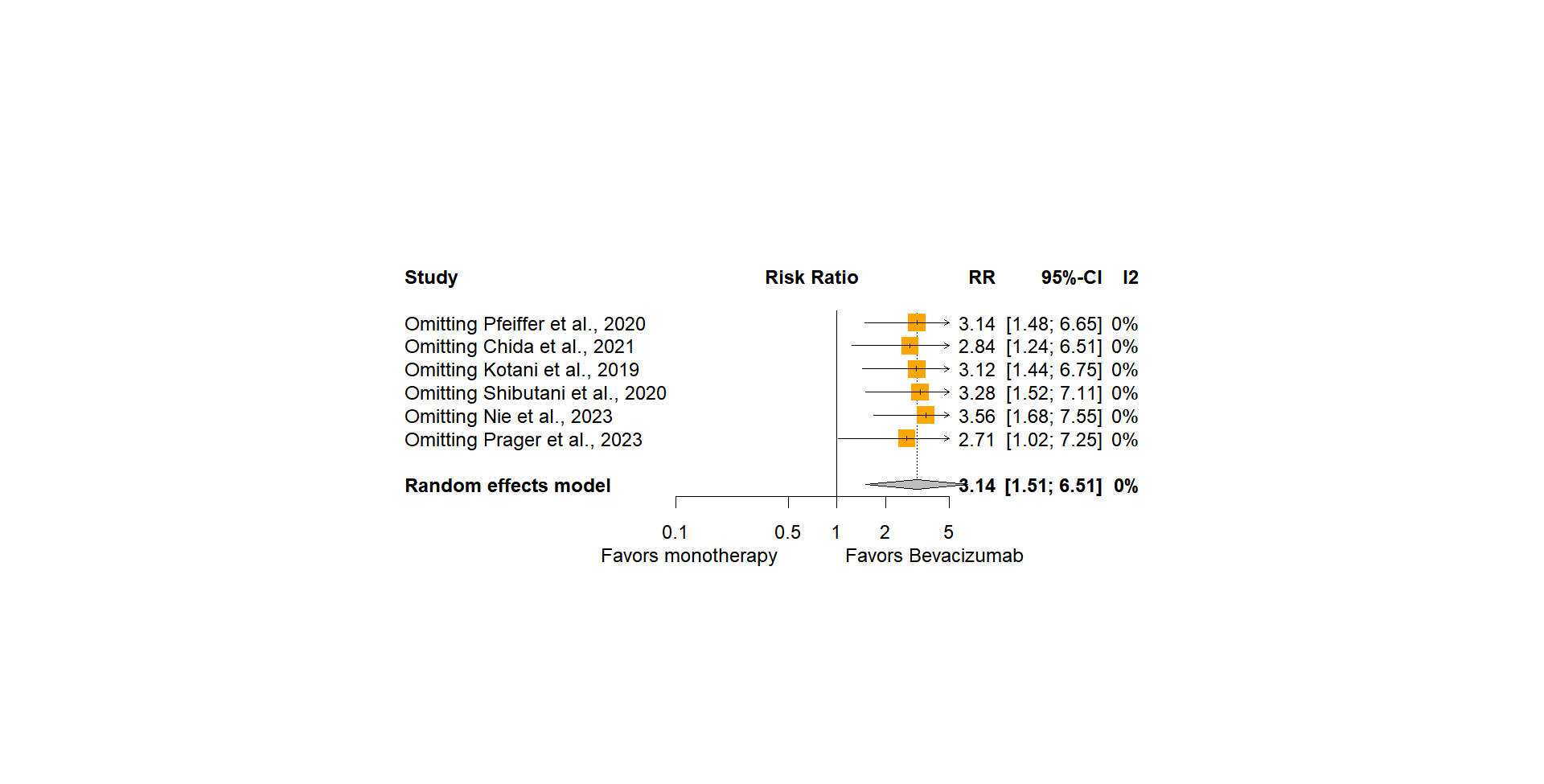


**D. Disease Control Rate (DCR)**

**
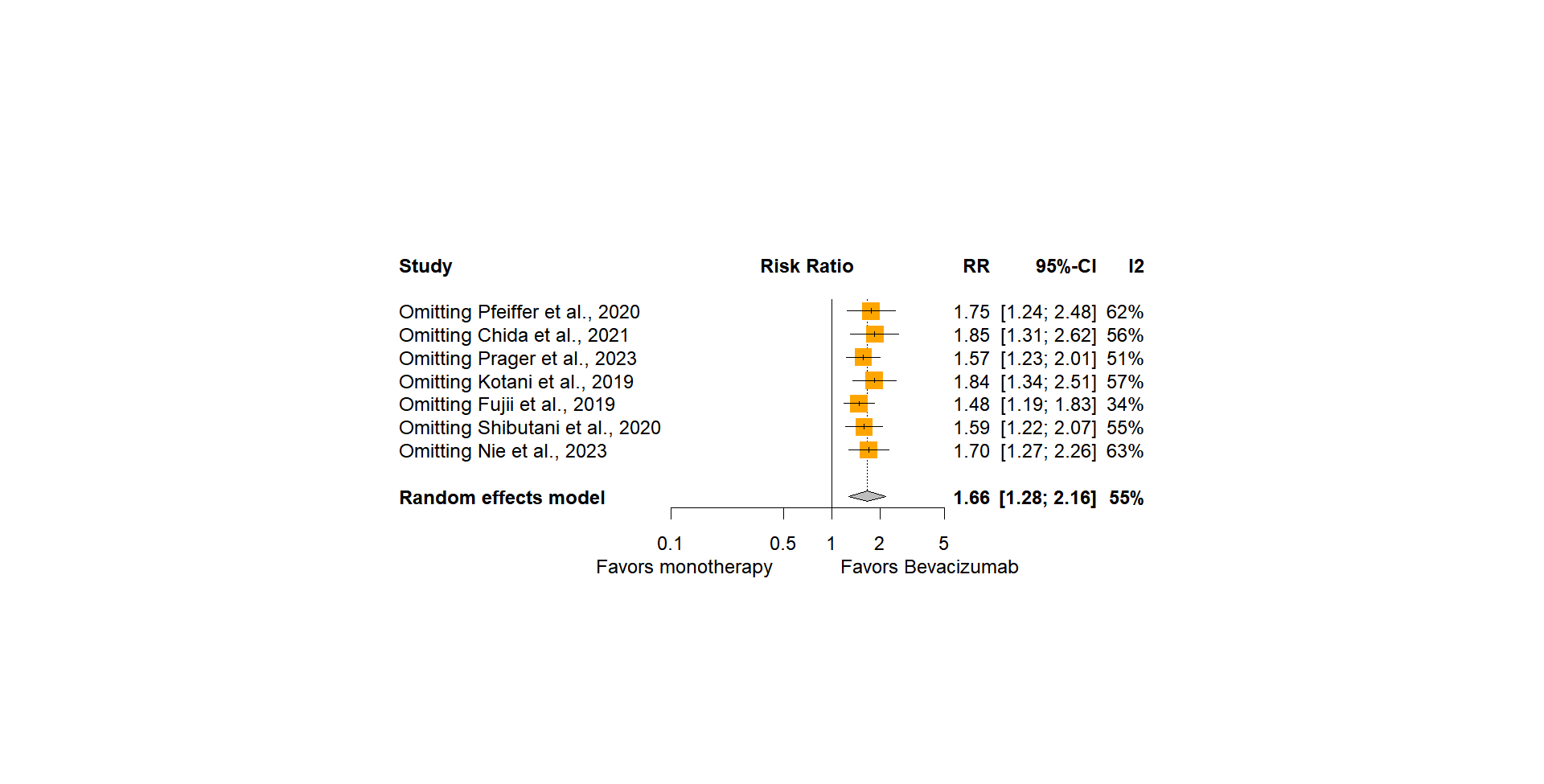
**

**Supplementary Figure 3** Heterogeneity analysis. **A.1.** Heterogeneity Overall Survivor**.** **A.2.** Funnel AKT Overall Survivor. **B.1.** Heterogeneity Progression-free survival. **B.2.** Heterogeneity Progression-free survival. **C.1.** Heterogeneity Objective response rate. **C.2.** Heterogeneity Objective response rate. **D.1.** Heterogeneity Disease control rate (DCR). **D.2.** Heterogeneity Disease control rate (DCR).

**A.1. Heterogeneity Overall Survivor**

**
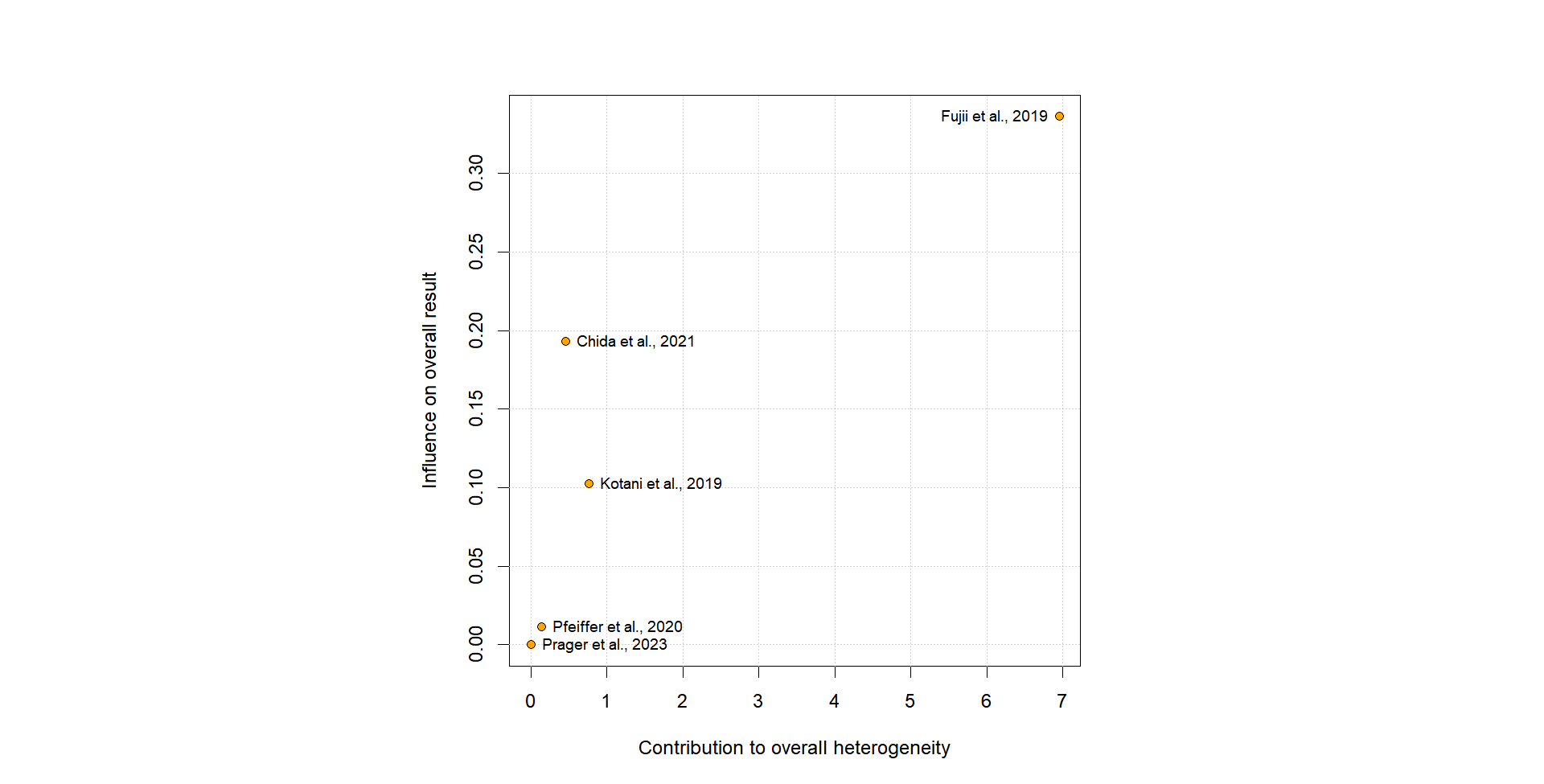
**

**A.2. Funnel Overall Survivor**

**
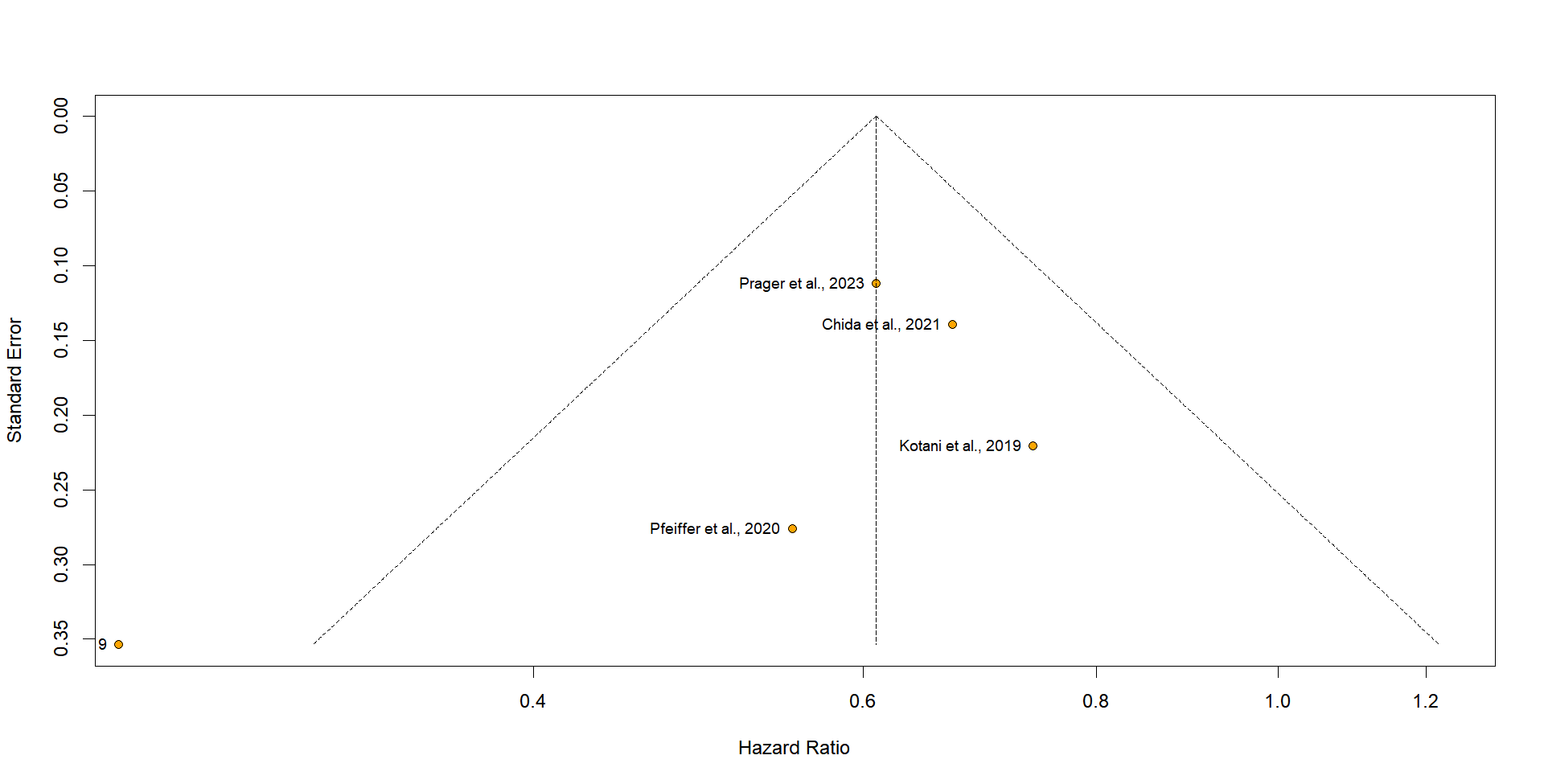
**

**B.1. Heterogeneity Progression-free survival**

**
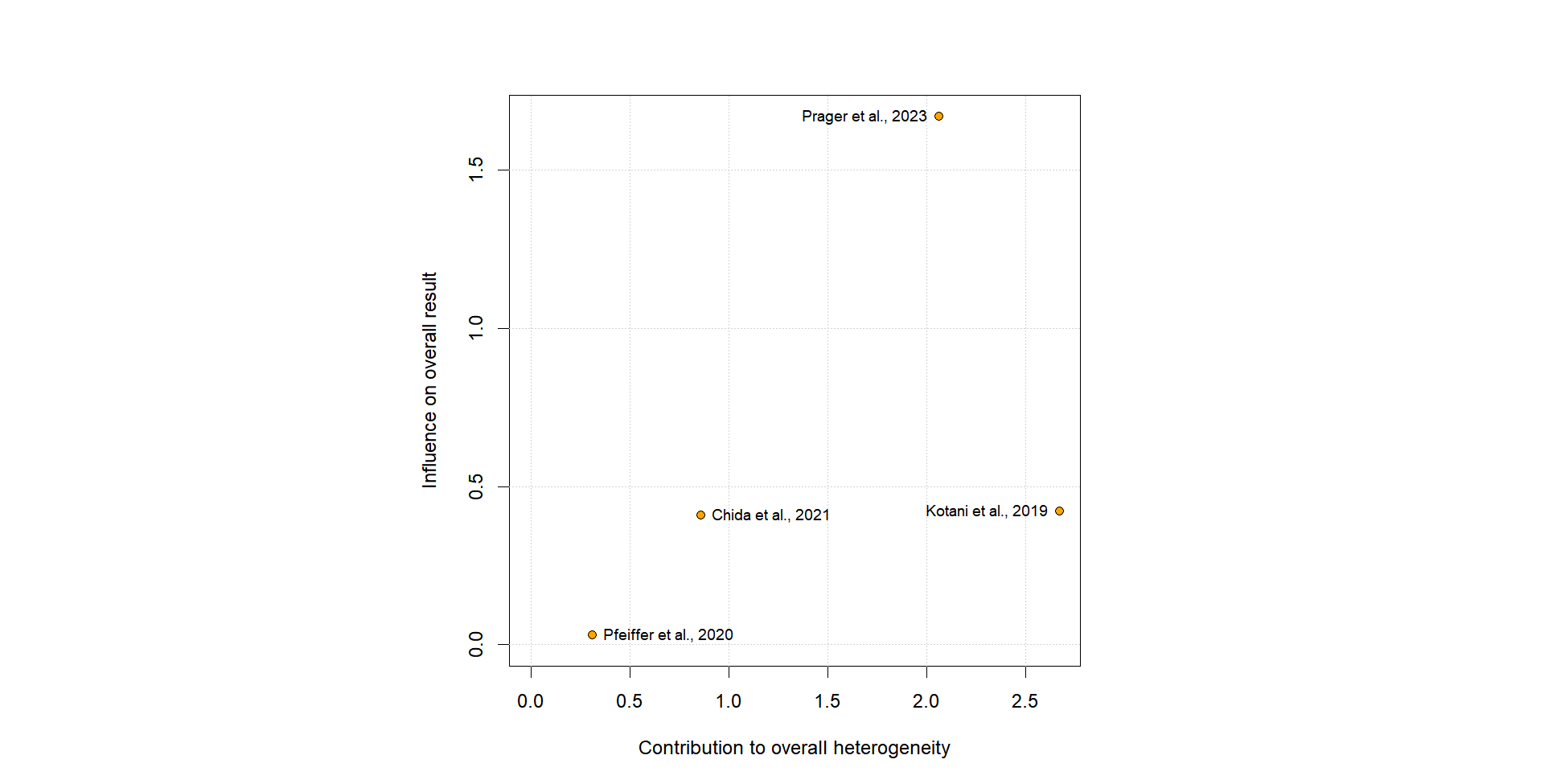
**

**B.2. Heterogeneity Progression-free survival**

**
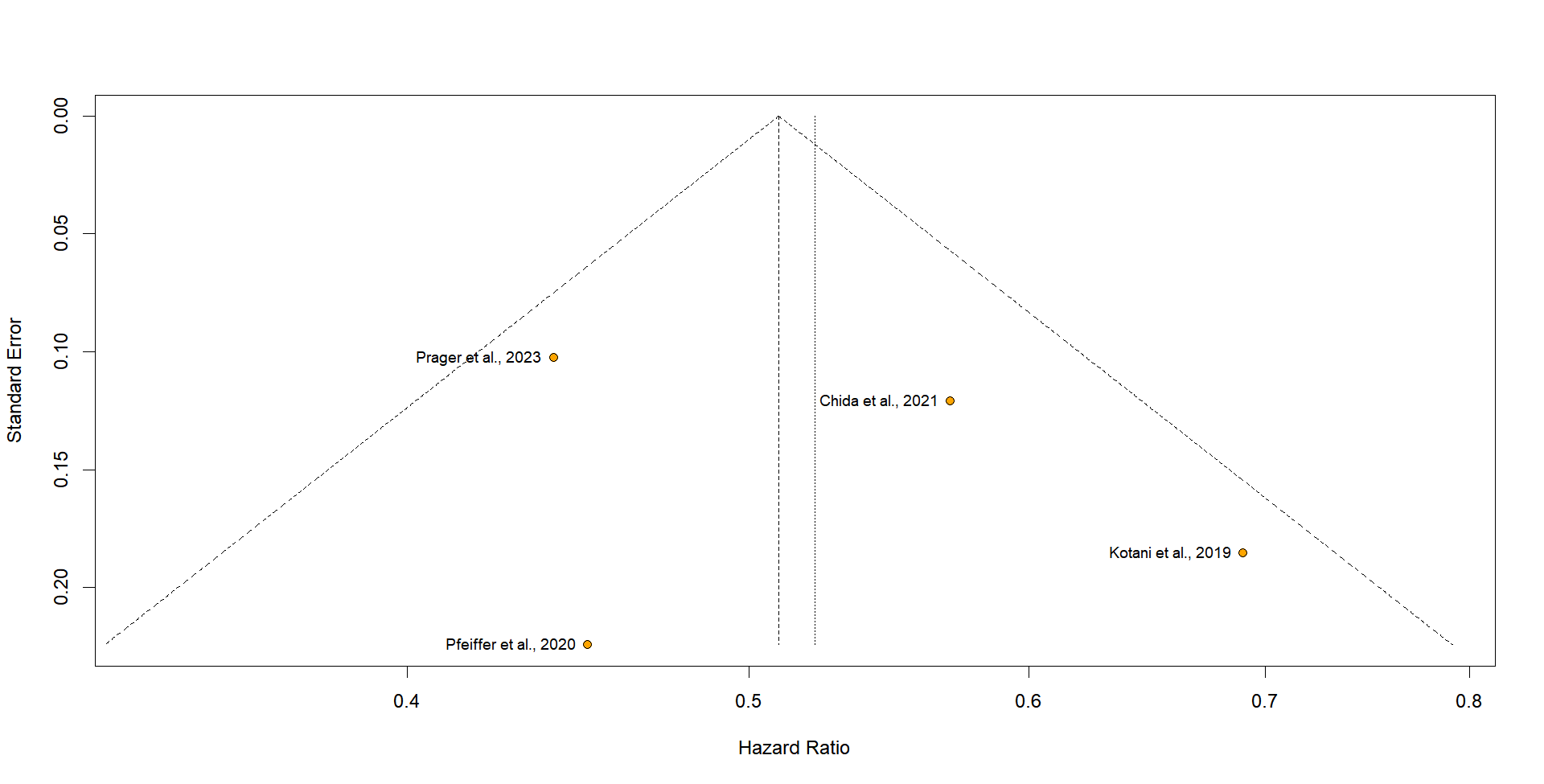
**

**C.1. Heterogeneity Objective response rate**

**
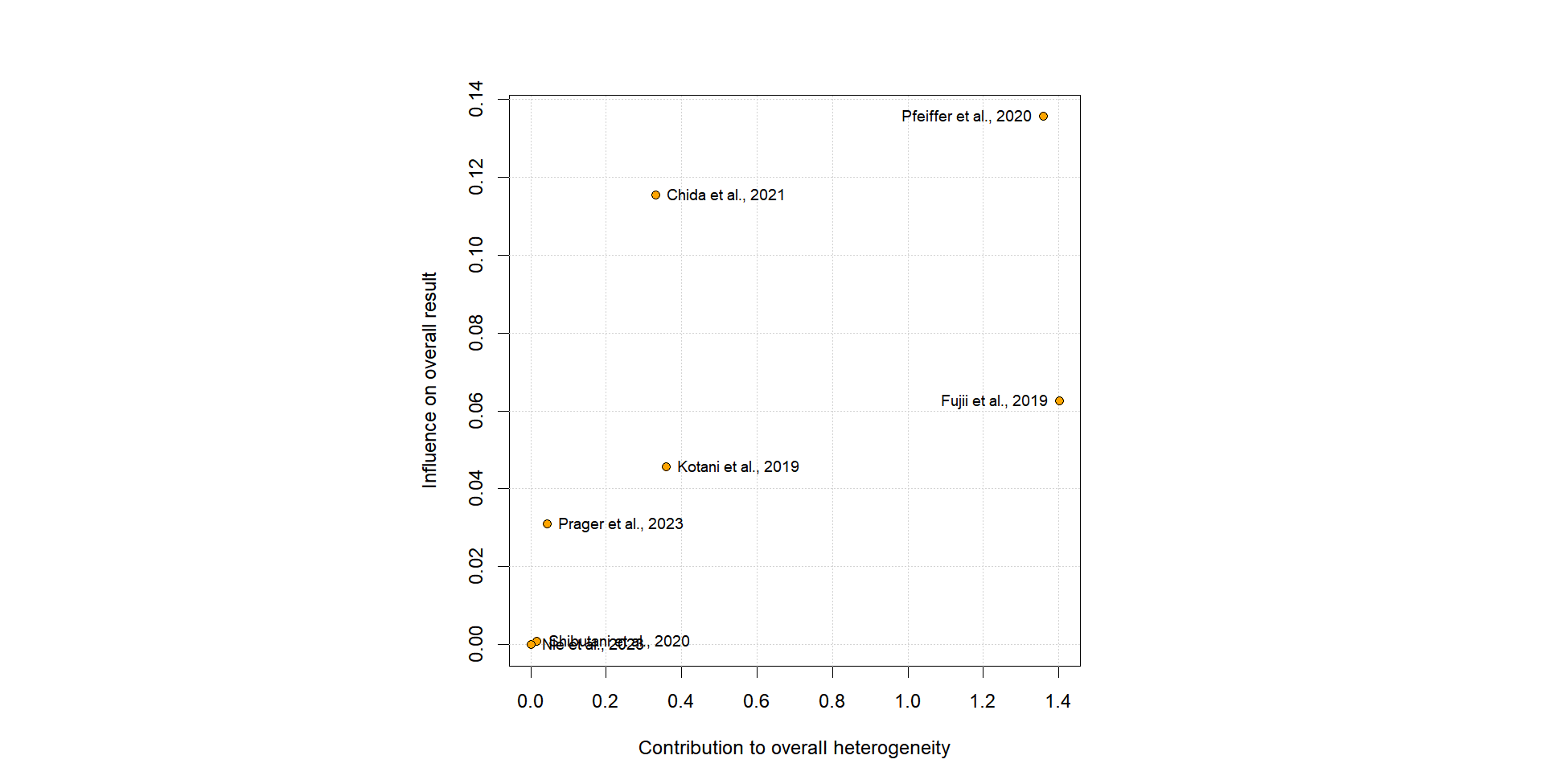
**

**C.2. Heterogeneity Objective response rate**

**
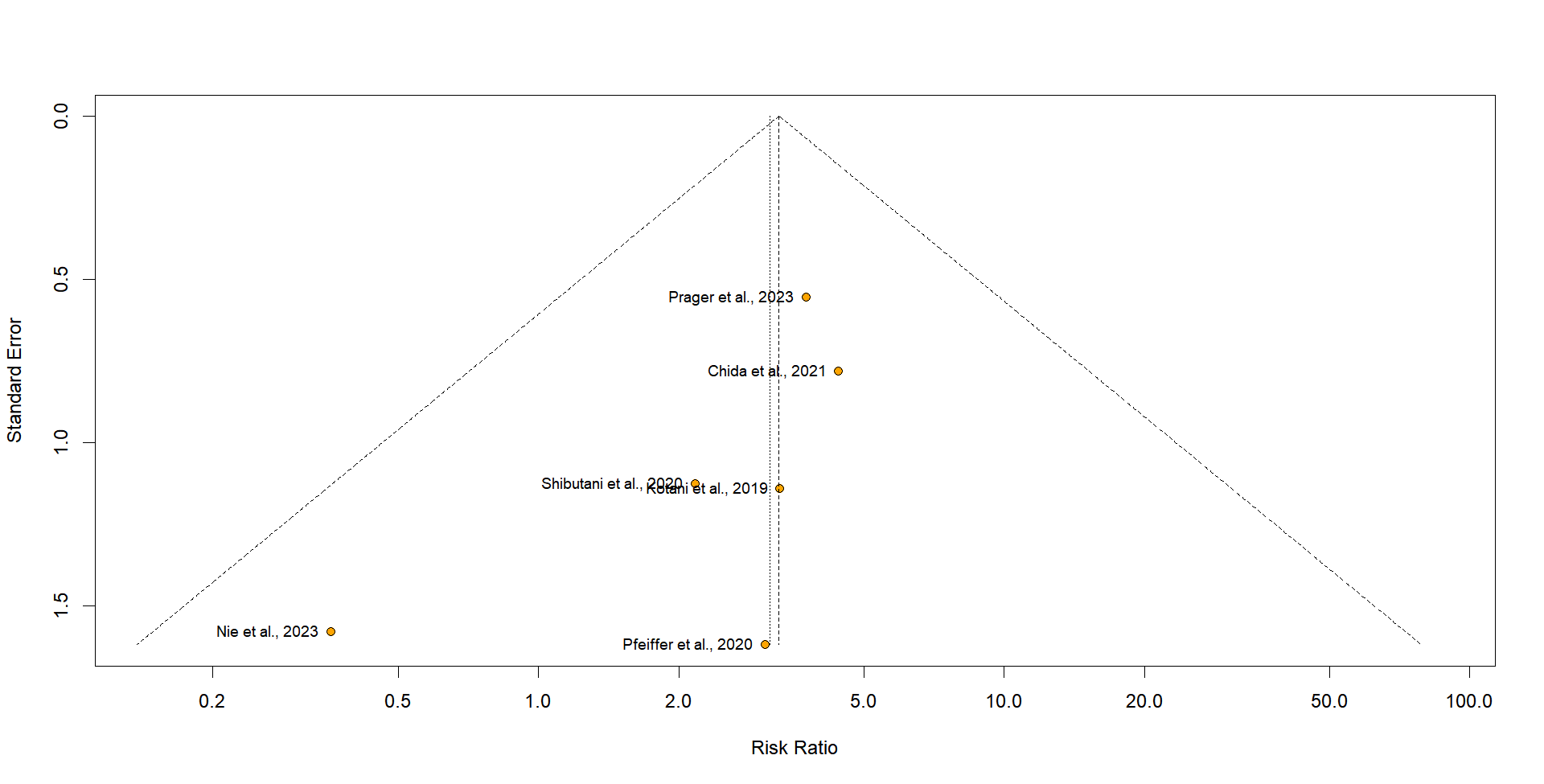
**

**D.1. Heterogeneity Disease control rate (DCR)**

**
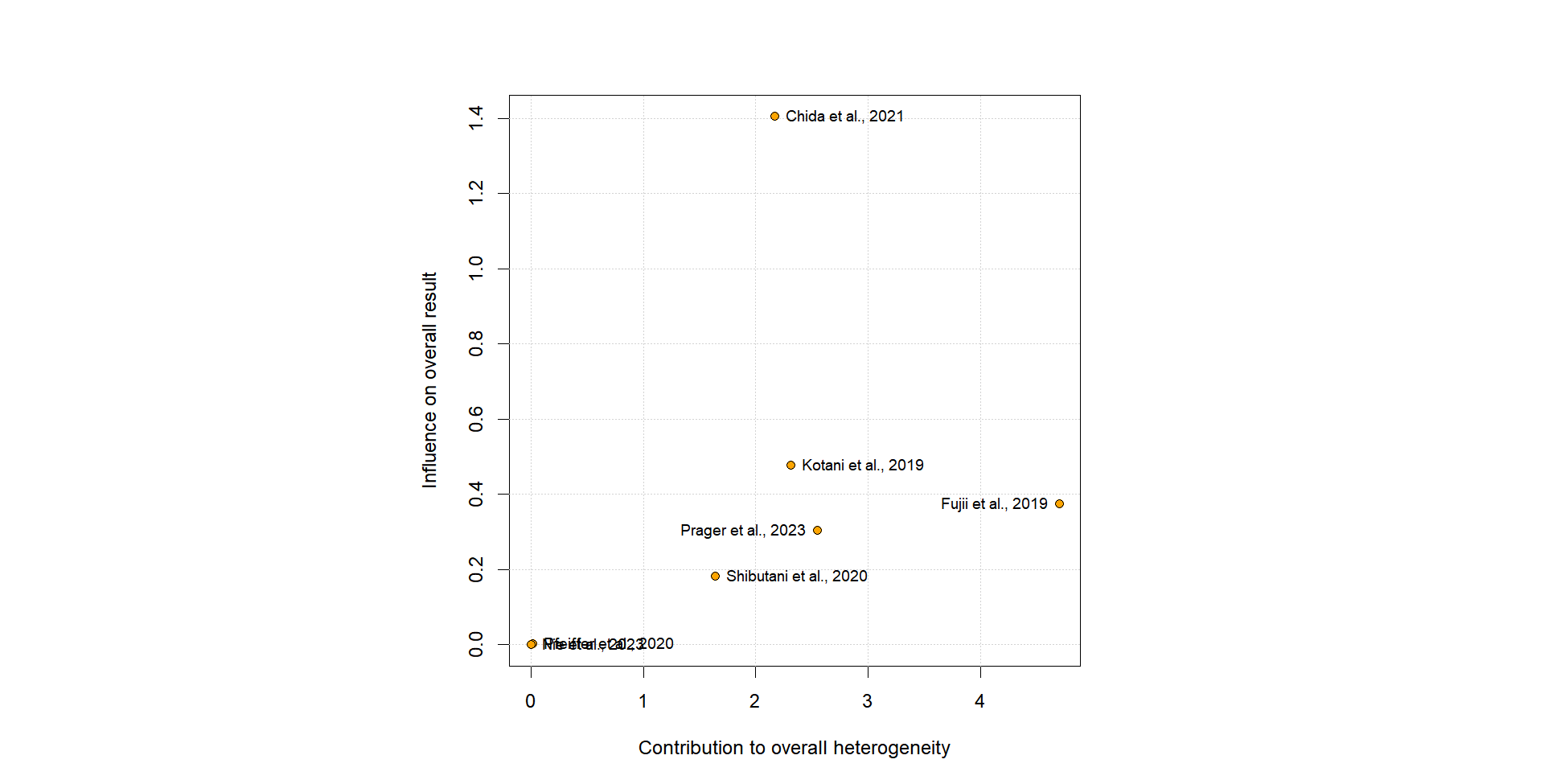
**

**D.2. Heterogeneity Disease control rate (DCR)**

**
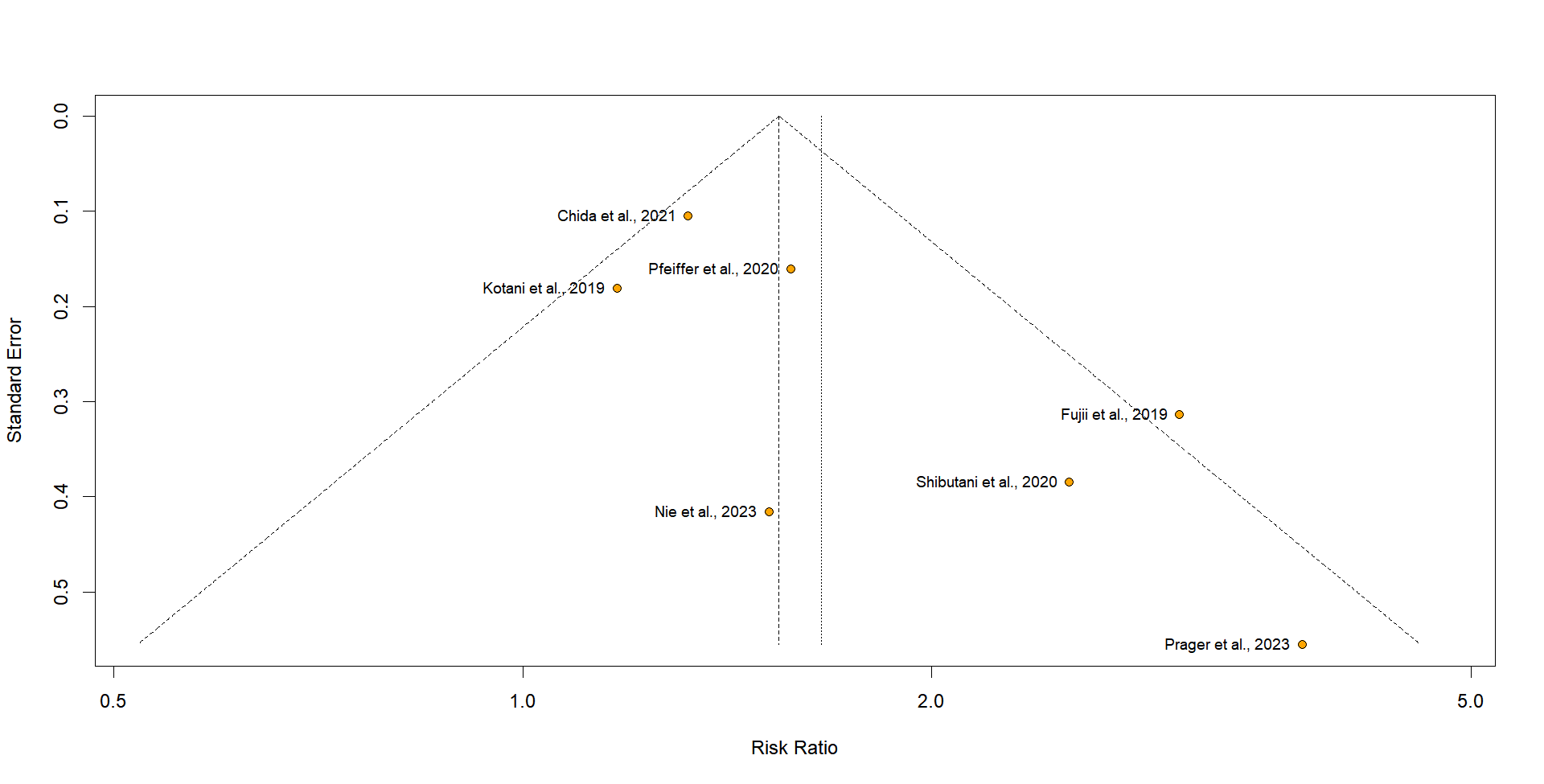
**

**Supplementary Figure 4** Methodological quality summary using RoB2. **A.** Risk of bias domains in Randomized clinical trials (RCTs). **B.** Overall risk of bias in Randomized clinical trials (RCTs). **C.** Risk of bias domains in Retrospective cohort studies (RCS). **D.** Overall risk of bias in Retrospective cohort studies (RCS)

**A.** **Risk of bias domains in Randomized clinical trials (RCTs)**

**
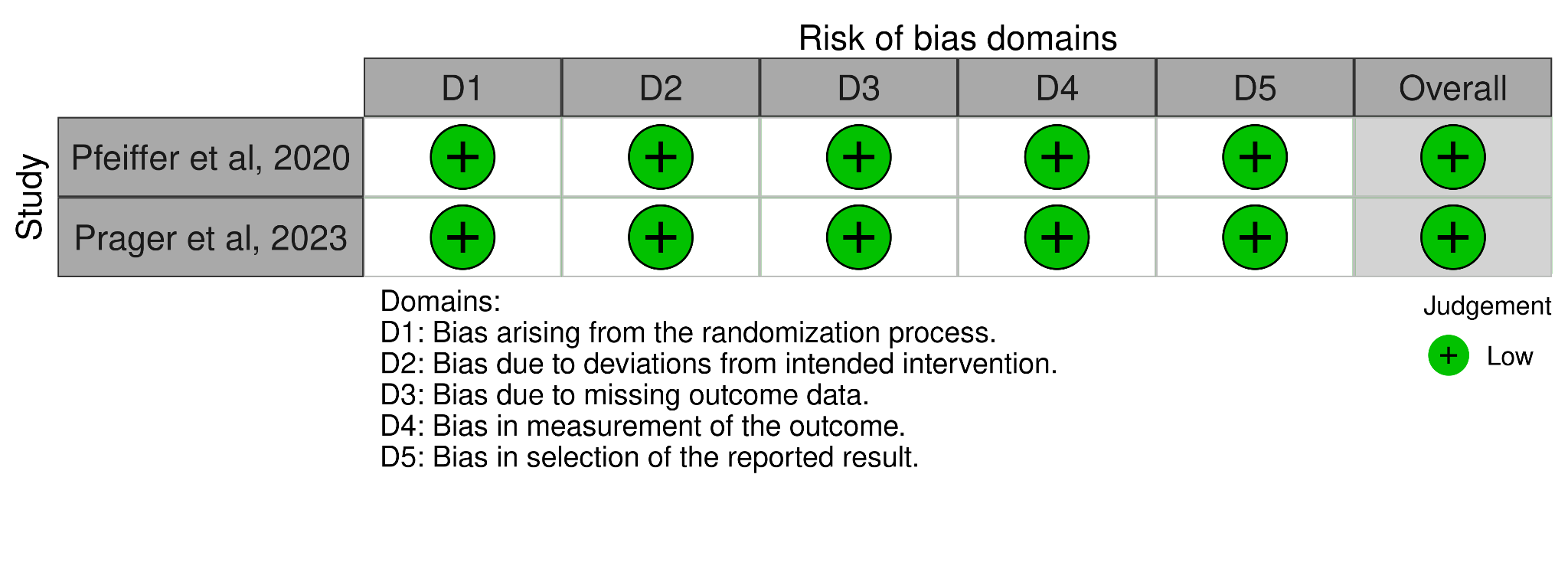
**

**B. Overall risk of bias in Randomized clinical trials (RCTs)**

**
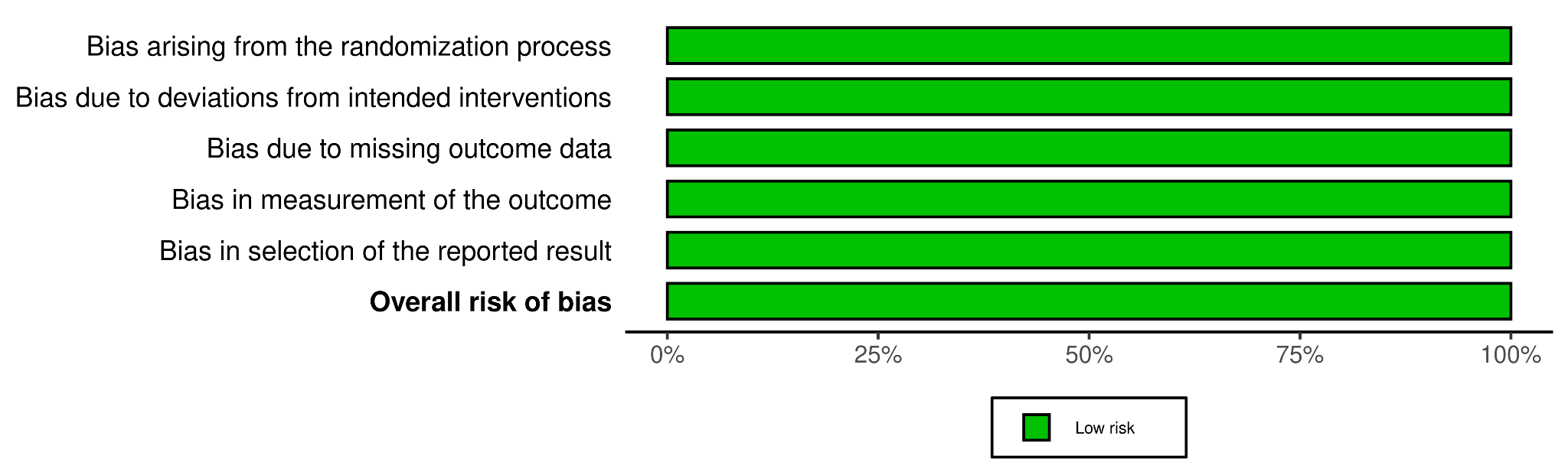
**

**C.** **Risk of bias domains in Retrospective cohort studies (RCS)**

**
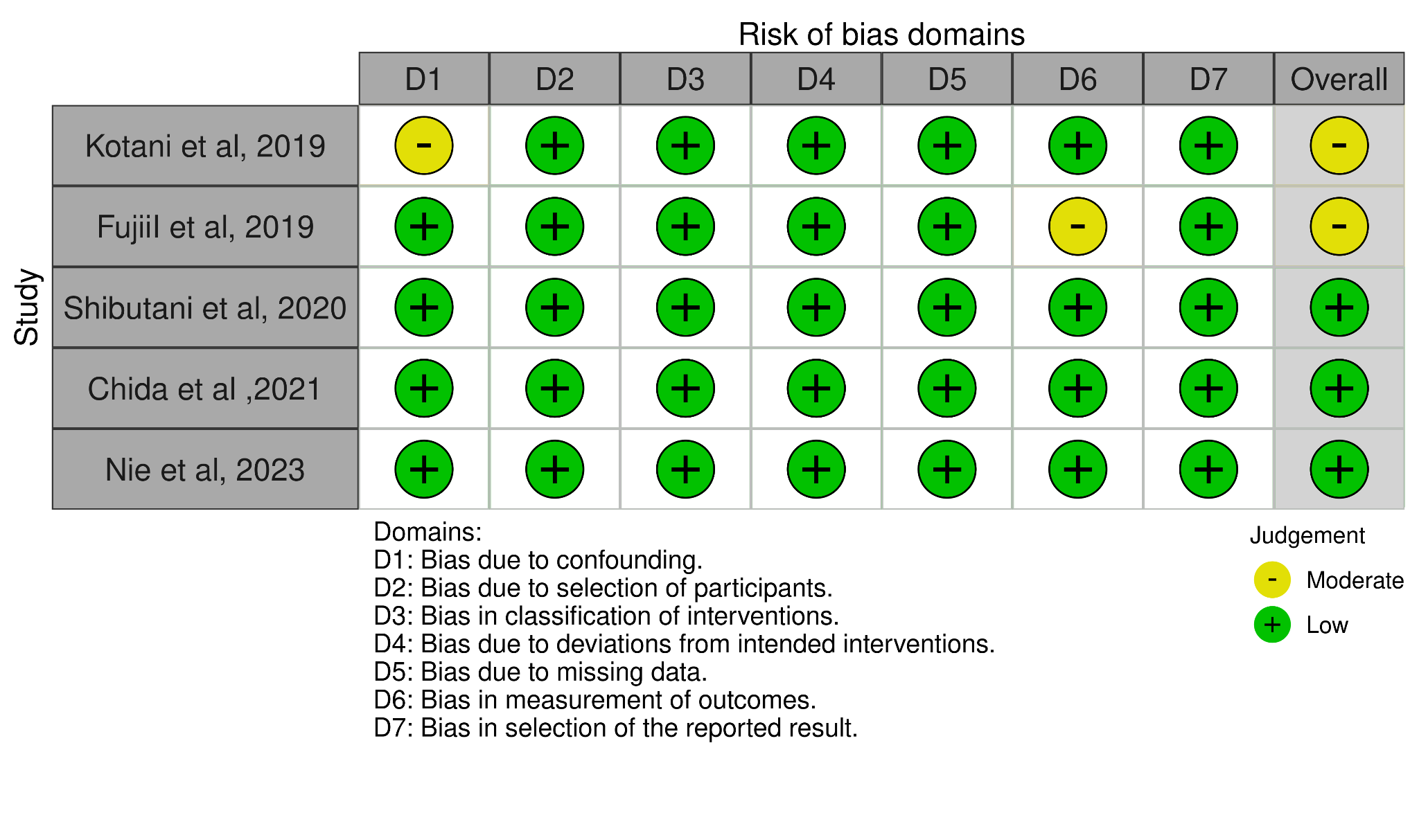
**

**D.** **Overall risk of bias in Retrospective cohort studies (RCS)**


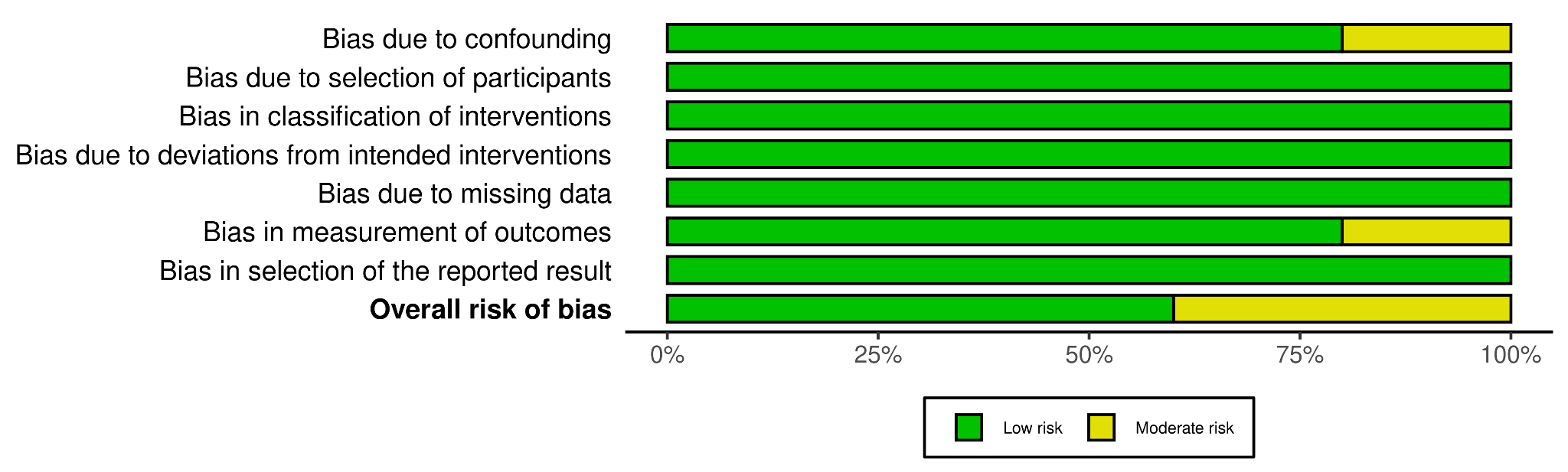

Supplement: Supplementary file 1 — Supplementary Material 1. [file 12885_2024_12447_MOESM1_ESM.docx]
